# MT1‐MMP‐Activated Liposomes to Improve Tumor Blood Perfusion and Drug Delivery for Enhanced Pancreatic Cancer Therapy

**DOI:** 10.1002/advs.201902746

**Published:** 2020-07-10

**Authors:** Yan Wei, Sha Song, Nianxiu Duan, Feng Wang, Yuxi Wang, Yiwei Yang, Chengyuan Peng, Junjun Li, Di Nie, Xinxin Zhang, Shiyan Guo, Chunliu Zhu, Miaorong Yu, Yong Gan

**Affiliations:** ^1^ Shanghai Institute of Materia Medica Chinese Academy of Sciences Shanghai 201203 China; ^2^ Department of Pharmacy Medical College of Nanchang University Nanchang 330066 China; ^3^ Department of Medicinal Chemistry Shanghai Hansoh Biomedical R&D Inc. Shanghai 201203 China; ^4^ State Key Laboratory of Drug Research Shanghai Institute of Materia Medica Chinese Academy of Sciences Shanghai 201203 China

**Keywords:** membrane type 1‐matrix metalloproteinase‐activated cilengitide, pancreatic cancer, smart liposomes, vascular promotion

## Abstract

Promoting tumor angiogenesis effectively and specifically to resolve tumor‐associated hypoperfusion holds promise for improving pancreatic cancer therapy. Herein, a doxorubicin (DOX) loaded smart liposome, MC‐T‐DOX, is constructed, that carries appropriately low‐density cilengitide, an *α*v*β*3 integrin‐specific Arg‐Gly‐Asp (RGD)‐mimetic cyclic peptide, via a membrane type 1‐matrix metalloproteinase (MT1‐MMP) cleavable peptide. After being administered systemically in a hypoperfused pancreatic cancer mouse model at a low dose of cilengitide, the proangiogenic activity of MC‐T‐DOX is specifically “turned on” in tumor vessels through cleavage by MT1‐MMP on tumor endothelial cells to release cilengitide. This locally released cilengitide increases tumor blood perfusion, thereby improving the accumulation and distribution of MC‐T‐DOX in the tumor site. The loaded‐DOX then displays enhanced penetration and increased cellular uptake upon heat‐triggered release from MC‐T‐DOX in the tumor interstitium, contributing to the improved tumor therapy efficacy. Therefore, the strategy of combining the modulation of tumor vascular promotion with smart nanodrug delivery represents a promising approach to improving drug delivery and therapeutic efficacy in a wide range of hypoperfused tumors.

## Graphical abstract

Low‐density membrane type 1‐matrix metalloproteinase (MT1‐MMP)‐activated cilengitide (MC) is modified onto thermosensitive liposomes loaded with doxorubicin (DOX), yielding MC‐T‐DOX. MC‐T‐DOX is activated by MT1‐MMP on tumor endothelial cells to release cilengitide, which then improves tumor blood perfusion, thereby enhancing intratumoral delivery of MC‐T‐DOX. Subsequently, in the interstitium, heat‐triggered DOX release from MC‐T‐DOX facilitates its bioavailability, thereby exerting improved antitumor efficacy.

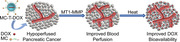

## 1 Introduction

Pancreatic cancer is currently the fourth leading cause of cancer‐related death in North America and Europe and has a less than 10% 5‐year overall survival rate for all stages.^[^ ^1^, ^2^ ^]^ One of the major predicaments for the treatment of pancreatic cancer is its sparse and diminished functional tumor vasculature,^[^ ^3^, ^4^, ^5^ ^]^ which leads to the impaired tumor blood perfusion limiting the access of chemotherapeutics, molecular targeted biologics, or nanomedicines to tumor cells. Diminishing the dense extracellular matrix in pancreatic cancer has been reported to re‐expand the tumor blood vessels and increase intratumoral drug delivery.^[^ ^5^, ^6^, ^7^, ^8^ ^]^ However, uncontrollable depletion of the stroma would result in unexpected risks, including promoting tumor invasiveness and even metastasis.^[^ ^9^, ^10^ ^]^ Vascular promotion, recruiting new blood vessels into previously unperfused regions, therefore, may be a preferable modulation strategy.^[^ ^11^, ^12^ ^]^

Integrins, the family of cell surface extracellular matrix receptors, can promote endothelial cell migration and survival, both of which are essential features of angiogenesis.^[^ ^13^ ^]^ It is well known that micromolar concentrations of cilengitide, an *α*v*β*3 integrin‐specific Arg‐Gly‐Asp (RGD)‐mimetic cyclic peptide, exert highly antiangiogenic effect and induce apoptosis of growing endothelial cells via blocking the interaction of *α*v*β*3 with its extracellular matrix ligands.^[^ ^14^, ^15^ ^]^ In contrast, recent reports have revealed that nanomolar concentrations of cilengitide can actually enhance tumor angiogenesis by altering the endothelial growth factor receptor‐2 trafficking to promote endothelial cell migration.^[^ ^12^ ^]^ It should be noted that this modulation would reduce tumor metastasis, thereby representing an appealing strategy to resolve tumor‐associated hypoperfusion.^[^ ^11^, ^12^ ^]^ However, cilengitide in circulation would suffer from rapid elimination.^[^ ^16^ ^]^ Conjugating RGD peptides to nanocarriers is a general strategy to prolong their circulation time. Nevertheless, a high number of RGD peptide modification might specifically address drugs to angiogenic endothelial cells because multivalent RGD‐ligands facilitate higher affinity and internalization,^[^ ^17^ ^]^ thereby destroying the tumor vasculature.^[^ ^18^ ^]^ An appropriately low density modification, on the other hand, would avoid *α*v*β*3‐mediated endocytosis, but resulting in the loss of cilengitide activity. That is because the formation of focal contacts is necessary for cell migration, which is favored by higher RGD surface densities.^[^ ^19^ ^]^ We hence hypothesize that designing nanocarriers modified with appropriately low‐density cilengitide through a cleavable linker, which can acquire a prolonged circulation time and selectively release cilengitide to regain its activity in the tumor vessels, might be a promising strategy to resolve tumor‐associated hypoperfusion and improve pancreatic cancer therapy.

**Scheme 1:**
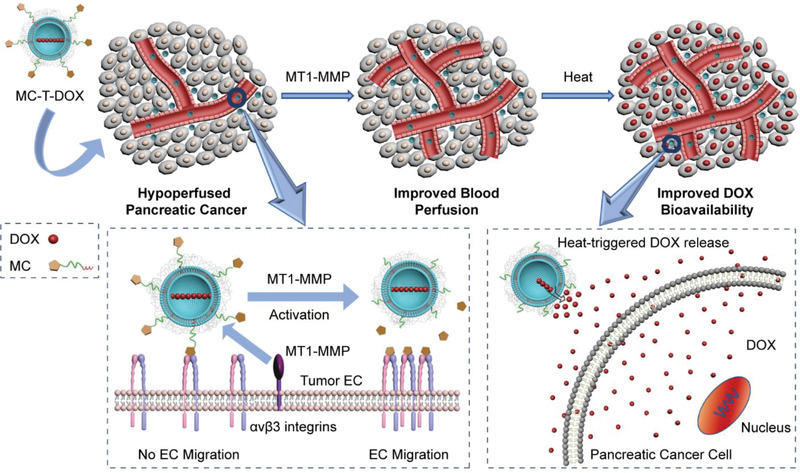
Mode of action by which MC‐T‐DOX effectively and specifically improves tumor blood perfusion and drug delivery in pancreatic cancer. Membrane type 1‐matrix metalloproteinase (MT1‐MMP)‐activated cilengitide (MC) at an appropriately low density is modified onto thermosensitive liposomes (TSLs) loaded with doxorubicin (DOX), yielding MC‐T‐DOX. When MC‐T‐DOX was injected intravenously into the mice bearing hypoperfused pancreatic tumor at a low dose of cilengitide, it might be activated by MT1‐MMP on tumor endothelial cells (ECs) to release cilengitide, which then promotes EC migration and angiogenesis, thereby resulting in improved accumulation and a wider distribution of MC‐T‐DOX in the tumor site. Subsequently, in the interstitium, heat‐triggered DOX release from MC‐T‐DOX facilitates its bioavailability, thereby exerting improved antitumor efficacy.

Membrane type 1‐matrix metalloproteinase (MT1‐MMP), an enzyme related to tumor angiogenesis, is highly expressed on tumor endothelial cells.^[^ ^20^, ^21^ ^]^ It can selectively cleave specific peptide sequences. Herein, we first designed an amphiphilic peptide by conjugating cilengitide to the C18 alkyl chain via a MT1‐MMP cleavable peptide (KRRQLGLPALS*β*Ala),^[^ ^22^ ^]^ named MT1‐MMP‐activated cilengitide (MC). Then, low‐density MC was loaded onto doxorubicin (DOX)‐loaded thermosensitive liposomes (TSLs), yielding MC‐T‐DOX (**Scheme** **1**). When administered systemically in a hypoperfused pancreatic cancer mouse model at a low dose of cilengitide, MC‐T‐DOX readily acquired prolonged circulation time; its proangiogenic activity was specifically “turned on” in tumor vessels through cleavage by MT1‐MMP on tumor endothelial cells to release cilengitide. This locally released cilengitide enhanced tumor blood perfusion, improving the accumulation and distribution of MC‐T‐DOX in the tumor site. DOX then exhibited improved bioavailability after heat‐triggered release from MC‐T‐DOX, and thus significantly inhibited tumor growth.

## 2 Results and Discussion

### 2.1 Preparation and Characterization of MC‐T‐DOX

**Figure 1:**
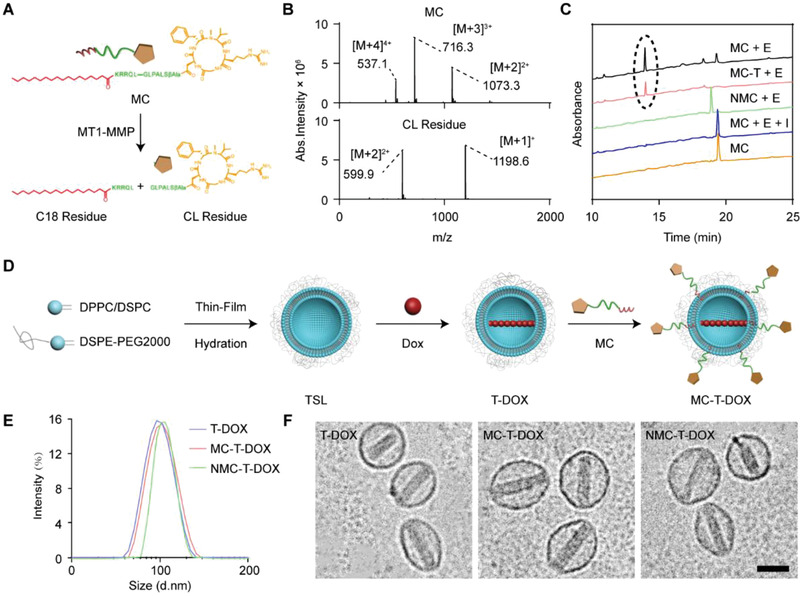
Preparation and characterization of MC‐T‐DOX. A) Schematic illustration of MC cleavage by MT1‐MMP to release cilengitide. B) MS spectrum of (upper row) MC (MW: 2145.3) and (lower row) the degradation peak (cilengitide (CL) residue; MW: 1197.6) C) HPLC chromatograms of MT1‐MMP‐mediated cleavage of cilengitide from MC. The black circle indicates the peak of cilengitide residue. “E” indicates membrane type 1‐matrix metalloproteinase. “I” indicates the matrix metalloproteinase inhibitor GM6001. D) Schematic illustration of the preparation procedure of MC‐T‐DOX. E) Size distribution and F) Cryo‐TEM images of T‐DOX, MC‐T‐DOX and NMC‐T‐DOX. Scale bar: 100 nm.

MT1‐MMP is highly expressed on tumor endothelial cells (ECs).^[^ ^20^, ^21^ ^]^ Therefore, we selected MT1‐MMP as an endogenous proteinase to activate MC‐T‐DOX. To prepare MC‐T‐DOX, we first synthesized MT1‐MMP‐activated cilengitide (MC) by standard Fmoc chemistry protocols (Scheme S1, Supporting Information), which was expected to release cilengitide after MT1‐MMP cleavage (**Figure** **1**A). Successful synthesis of MC was confirmed by the identification of three multiply‐charged ion peaks in a spectrum obtained by mass spectrometry (MS) (Figure 1B, upper row) and its purity was >98% as determined by HPLC (Figure S1, Supporting Information). An MT1‐MMP insensitive analog of MC, C18‐krrqlglpals*β*ala‐cilengitide (NMC) was also synthesized using the same method as the negative control (Figure S2, Supporting Information).

To evaluate MT1‐MMP‐mediated cleavage of MC, MC was incubated with MT1‐MMP for 2 h with or without the broad‐spectrum MMP inhibitor GM6001, and then the solution was analyzed using HPLC‐UV/MS. The peak of MC (*t*_R_: 19.3 min) almost disappeared postincubation with MT1‐MMP, and this was accompanied by the appearance of a degradation peak (*t*_R_: 13.8 min) (Figure 1C). The identity of this degradation product was confirmed as cilengitide residue by molecular ion peaks in the MS spectrum (Figure 1B, lower row), corresponding to the sizes of the predicted digested fragments (Figure 1A). In contrast, in the presence of the MMP inhibitor GM6001, MC cleavage was completely inhibited. In addition, NMC was not cleaved by MT1‐MMP, confirming its insensitivity (Figure 1C). These data suggest that MC can be cleaved by MT1‐MMP to release free cilengitide.

The preparation of MC‐T‐DOX is illustrated in Figure 1D. First, we prepared blank TSLs via thin‐film hydration and stepwise extrusion. Then, DOX was encapsulated into these TSLs via an ammonium sulfate gradient method, to yield T‐DOX. T‐DOX achieved a high DOX encapsulation efficiency of 87.2% (Table S1, Supporting Information). To prepare MC‐T‐DOX, low‐concentration MC was located on the outer leaflet of T‐DOX via the postinsertion method,^[^ ^23^ ^]^ which would facilitate its interaction with MT1‐MMP. MC achieved a drug loading efficiency of 0.021 mol% (with respect to phospholipid) through the interaction of its hydrophobic tails with lipid bilayer. It was calculated that 0.09 pmol cm^−2^ cilengitide was present on the TSL surfaces, based on the estimation that 80 000 phospholipid molecules form one liposome of ≈100 nm.^[^ ^24^ ^]^ This density was lower by more than 200‐fold than the density that was previously reported to trigger *α*v*β*3‐mediated endothelial uptake.^[^ ^18^ ^]^ Comparable low‐density NMC (0.020 mol%) was successfully postinserted into T‐DOX to yield NMC‐T‐DOX, serving as a MT1‐MMP insensitive control. MC‐T‐DOX and NMC‐T‐DOX achieved a comparable DOX encapsulation efficiency (86.1% and 85.8%, respectively) to that of T‐DOX (Table S1, Supporting Information), indicating that incubation of T‐DOX with MC or NMC at 37 °C did not cause DOX leakage. The hydrodynamic diameters of prepared nanovesicles were all ≈100 nm (Figure 1E), consistent with the Cryo transmission electron microscope (Cryo‐TEM) results (Figure 1F). We note that MC‐T‐DOX or NMC‐T‐DOX displayed a slight increase in size (≈8 nm) relative to T‐DOX (Figure 1E and Table S1, Supporting Information), due to the presence of low‐density cilengitide on their surface.

To examine MT1‐MMP‐mediated release of cilengitide from TSL surface, we incubated MC‐T (MC containing TSLs) with MT1‐MMP for 2 h and the supernatant was analyzed with HPLC. As shown in Figure 1C, a peak at a *t*_R_ of 13.8 min appeared, which was in good agreement with cilengitide residue. Therefore, cilengitide could be effectively released from MC‐T through MT1‐MMP cleavage.

Next, we examined the thermosensitivity of MC‐T‐DOX, which may be a crucial feature for improving DOX bioavailability. The thermosensitivity of TSLs depends on their lipid components.^[^ ^25^ ^]^ Hence, we first examined the effect of MC incorporation on the phase‐transition behavior of MC‐T‐DOX using differential scanning calorimetry. The phase‐transition temperature (*T_m_*) of TSLs slightly decreased from 45.26 to 44.24 °C post‐MC incorporation (Figure S3, Supporting Information), indicating that the thermosensitivity of MC‐T‐DOX was not impeded with MC. Next, the temperature‐dependent DOX release profile was examined in HEPES (N‐2‐hydroxyethylpiperazine‐N‐2‐ethane sulfonic acid) containing 90% fetal bovine serum. Consistent with Figure S3, Supporting Information, the release of DOX by both MC‐T‐DOX and T‐DOX displayed a clear temperature‐dependence, with less than 10% of DOX released at 37 °C (normal body temperature) but ≈80% released at 42 °C. Moreover, preincubation with MT1‐MMP did not change DOX release profile from MC‐T‐DOX at both temperatures, indicating that detachment of MC had a negligible effect on the thermosensitivity of MC‐T‐DOX (Figure S4, Supporting Information). These results suggest that MC‐T‐DOX could retain most drugs in circulation and rapidly release drugs under the tumor local heat.

Generally, due to the rapid blood passage through the tumor tissues, an ultrafast release profile of TSL formulation (<1 min) is needed to achieve intravascular drug release under heat. However, this release approach might cause tumor vascular injury.^[^ ^25^ ^]^ Herein, we used TSLs with a low DPPC content (56%) to increase the *T_m_* to ≈45 °C, thereby obtaining a relatively slower DOX release profile (over 1 h) at 42 °C. This relatively slow release TSL formulation would release DOX only in the interstitium under tumor local heat, thereby avoiding endothelial injury.^[^ ^26^ ^]^

### 2.2 DOX Bioavailability of MC‐T‐DOX

**Figure 2:**
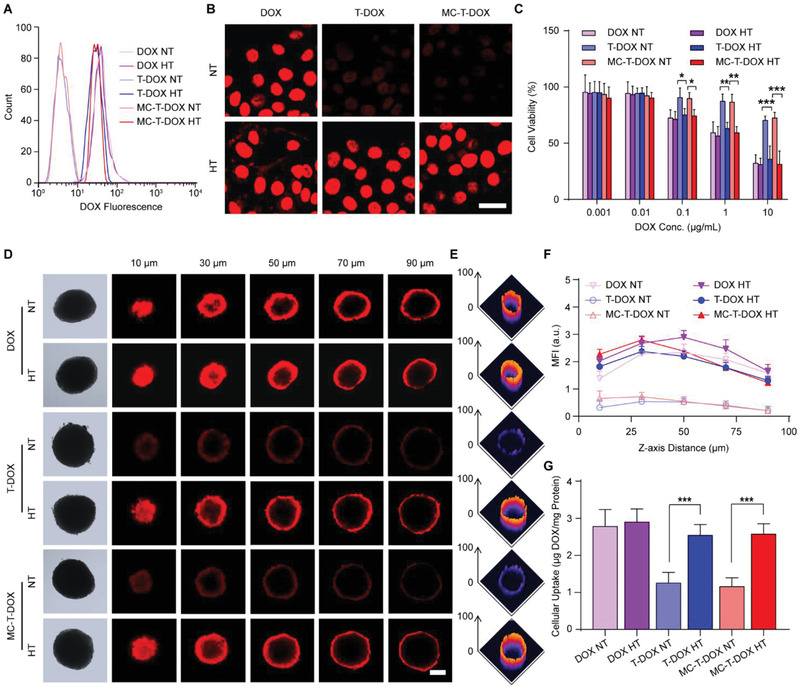
DOX bioavailability evaluation of MC‐T‐DOX. The formulations were subjected to pretreatment of mild hyperthermia (HT) (42 °C) or normothermia (NT) (37 °C) for 1 h before the experiment. A) Representative flow cytometry histograms and B) confocal images of cellular uptake of DOX in the indicated formulations by BxPC‐3. Scale bar: 20 µm. C) BxPC‐3 cells were incubated with the indicated formulations for 24 h and the cytotoxicity was measured through MTT assay (*n* = 6). D) DOX fluorescence distribution within the spheroids for the indicated formulations with HT or NT, which is also converted to E) surface plots above a depth of 50 µm using ImageJ. Scale bar: 200 µm. F) DOX fluorescence intensity versus the *Z*‐axis distance for the indicated formulations with HT or NT, wherein the surface of the spheroids was defined as 0 µm. G) Spheroid cellular uptake of DOX for the indicated formulations with HT or NT expressed as µg DOX per milligram protein (*n* = 3). Data are presented as the mean ± SD. *p < 0.05, **p < 0.01, ***p < 0.001 among the marked groups using nonparametric two‐tailed analysis of variance.

Generally, PEGylated liposomes displayed poor cellular uptake due to weak liposome‐cell interactions. Therefore, only released drugs from liposomes are active to tumor cells. For traditional liposomes such as Doxil, slow and incomplete drug release in the interstitium often limits drug bioavailability. The good thermosensitivity of MC‐T‐DOX motivated us to investigate whether heat‐triggered DOX release facilitates its bioavailability. First, we examined DOX uptake into BxPC‐3 cells. After pretreatment with mild hyperthermia (42 °C) or normothermia (37 °C) for 1 h, DOX, T‐DOX, or MC‐T‐DOX were incubated with BxPC‐3 cells and then DOX fluorescence was monitored. Flow cytometric examination revealed that free DOX showed good uptake regardless of hyperthermia or normothermia. Significantly higher DOX fluorescence intensity was detected in T‐DOX or MC‐T‐DOX with hyperthermia relative to normothermia (**Figure** **2**A and Figure S5, Supporting Information). This result suggests that the released free DOX readily produced significantly improved cellular uptake relative to liposomal DOX. Confocal images, acquired by confocal laser scanning microscope (CLSM), also displayed that T‐DOX or MC‐T‐DOX with hyperthermia exerted markedly stronger red fluorescence within the cellular nuclear areas than T‐DOX or MC‐T‐DOX with normothermia (Figure 2B). DOX could interfere with DNA replication and RNA synthesis within the cell nuclei, causing cell death. Hence, heat‐triggered release from liposomes could promote DOX accumulation at its intracellular target site.

Next, after pretreatment as described above, these formulations were incubated with BxPC‐3 cells to examine their cytotoxicity by MTT assay (Figure 2C). T‐DOX and MC‐T‐DOX with hyperthermia both exhibited more profound cytotoxicity than that with normothermia, as DOX concentration ranged from 0.1 to 10 µg mL^−1^, due to the improved DOX uptake into cells. These results indicated that heat‐triggered DOX release from T‐DOX or MC‐T‐DOX effectively improved DOX delivery at its intracellular target site, thereby resulting in enhanced tumor growth inhibition.

However, cell uptake may not accurately reflect drug availability in vivo due to the difference between cell monolayer and solid tumor. In vitro tumor spheroids are a versatile 3D models to simulate the in vivo status of tumor due to their similarity in morphology and biological microenvironment to solid tumors. Therefore, we established an avascular BxPC‐3 containing 3D tumor spheroid model and observed the delivery of different formulations within it. After pretreatment as described above, these formulations were incubated with the spheroids for 1 h. Then, DOX fluorescence was monitored using CLSM Z‐stack scanning and the images were also converted to surface plots using ImageJ (Figure 2D,E). Free DOX showed good penetration regardless of hyperthermia or normothermia. Following T‐DOX and MC‐T‐DOX treatment with normothermia, the red fluorescence of DOX was poor and mostly on the periphery of the spheroids, indicating a limited penetration into tumor. This result could be ascribed to limited penetration ability of these approximately 100‐nm TSL nanovesicles in tumor as reported previously.^[^ ^27^ ^]^ In contrast, DOX in the T‐DOX and MC‐T‐DOX with hyperthermia exhibited significantly improved penetration, with a strong fluorescent signal observed not only peripherally but also inside the spheroids, even at a scanning depth of 30 µm (Figure 2D,E). Indeed, at each scanning depth, significantly higher fluorescence intensity was observed in T‐DOX and MC‐T‐DOX with hyperthermia relative to normothermia (Figure 2F). This result occurred mainly because these released DOX from T‐DOX and MC‐T‐DOX were able to diffuse from an area of high concentration on the spheroid periphery into an area of low concentration within the spheroid interior.

The robust penetration of DOX in T‐DOX and MC‐T‐DOX with hyperthermia endowed it with a high opportunity to reach more tumor cells to boost uptake. Then, the cellular uptake of DOX was further measured by analyzing DOX amount in cell lysate from these spheroids. Free DOX showed good uptake regardless of hyperthermia or normothermia. T‐DOX and MC‐T‐DOX with hyperthermia showed more than twofold higher DOX uptake than that with normothermia (Figure 2G). Taken together, these results suggest that in the tumor interstitium heat‐triggered DOX release from T‐DOX and MC‐T‐DOX significantly improved DOX delivery at all tumor depth and resulted in more robust DOX uptake into tumor cells, thereby effectively improving DOX bioavailability.

Despite improved DOX bioavailability in the interstitium, the impaired tumor blood perfusion still remained a main obstacle to drug delivery. It greatly limited access of liposomal nanovesicles to tumor and left a long intervascular interstitial distance that drugs must traverse to reach tumor cells. Hence, subsequently, we investigated if MC‐T‐DOX could effectively improve tumor blood perfusion to facilitate its tumor delivery.

### 2.3 Proangiogenic Effect of MC‐T‐DOX

Multivalent RGD‐ligands facilitate higher affinity and internalization while a single peptide will not trigger uptake efficiently.^[^ ^17^ ^]^ Before investigating the proangiogenic effect of MC‐T‐DOX, we first examined whether MC‐T‐DOX boosted *α*v*β*3‐mediated endocytosis, which could injure ECs. In addition to the high expression of integrin *α*v*β*3, as reported previously,^[^ ^18^ ^]^ human umbilical vein endothelial cells (HUVECs) also express MT1‐MMP as shown through western blot assays (Figure S6, Supporting Information). Therefore, HUVECs were chose as an in vitro tumor EC model.

First, we investigated if the low‐density cilengitide modification (0.02 mol%) boosted EC uptake of liposomes. To this end, HUVEC uptake of NMC‐T‐DOX, which was insensitive to MT1‐MMP cleavage, with low density (0.02 mol%) or high density (2 mol%) cilengitide modification was measured by monitoring DOX fluorescence signal (Figure S7, Supporting Information). Flow cytometric examination revealed that NMC‐T‐DOX with 2 mol% cilengitide modification displayed significantly higher intracellular accumulation than cilengitide free T‐DOX, while NMC‐T‐DOX with 0.02 mol% cilengitide modification only exerted comparable endocytosis to T‐DOX. However, in the presence of a 20‐fold molar excess of free cilengitide, intracellular uptake of NMC‐T‐DOX with 2 mol% cilengitide modification was decreased to a significantly lower level, indicating that this endocytosis was mediated by the interaction between cilengitide and integrin *α*v*β*3. These findings suggest that low‐density (0.02 mol%) cilengitide modification could not boost EC uptake of liposomes efficiently.

**Figure 3:**
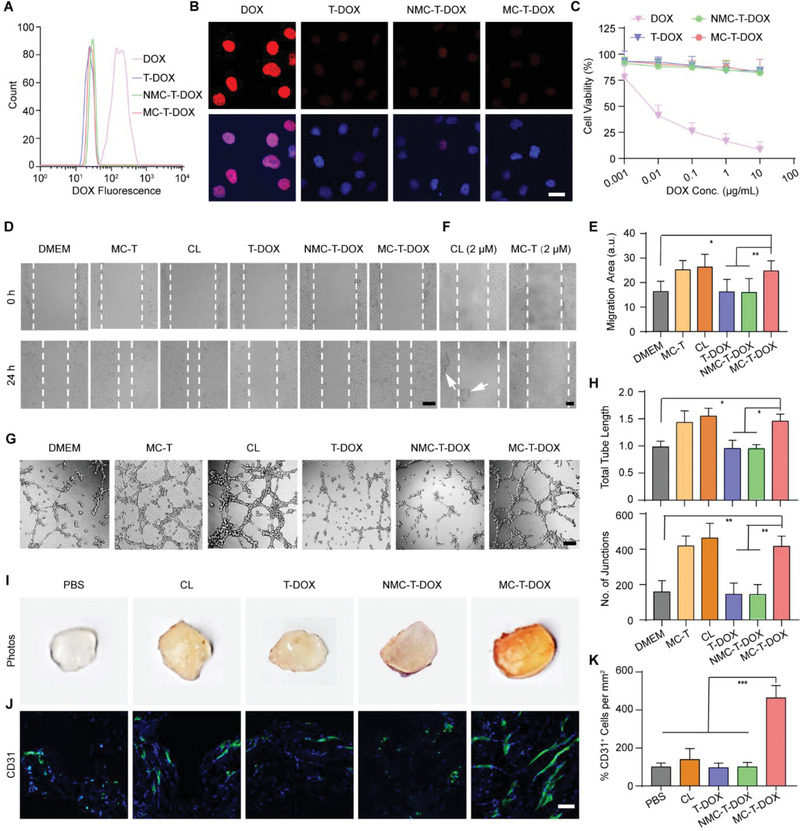
Proangiogenic effect of MC‐T‐DOX. A) Representative flow cytometry histograms and B) confocal images of cell uptake of DOX for the indicated formulations by HUVECs. Scale bar: 20 µm. C) HUVECs were incubated with the indicated formulations for 24 h and the cytotoxicity was measured through MTT assay (*n* = 6). D) Representative micrographs of HUVEC migration at 0 and 24 h at an identical cilengitide concentration of 2 nm and E) quantitative analysis after 24 h of migration measured as migration area (*n* = 8). Scale bar: 200 µm. F) Representative micrographs of HUVEC migration at 0 and 24 h at an identical cilengitide concentration of 2 µm. White arrows indicate the detached HUVEC clusters. Scale bar: 200 µm. G) Representative tube‐like structures and H) quantitative analysis of average number of junctions and total tube length after 8 h of incubation at an identical cilengitide concentration of 2 nm (*n* = 8). Scale bar: 200 µm. I) Representative macroscale photographs and J) anti‐CD31 antibody immunofluorescence staining (blue: cell nuclei; green: anti‐CD31 antibody) of Matrigel plugs on posttreatment day 12. Scale bar: 50 µm. K) Percentage of CD31^+^ ECs per mm^2^ in each group relative to that in the PBS group (*n* = 8). CL: cilengitide. Data are presented as mean ± SD. *p < 0.05, **p < 0.01, ***p < 0.001 among the marked groups using nonparametric two‐tailed analysis of variance.

Then, we further investigated EC uptake of MC‐T‐DOX. Flow cytometric examination showed that MC‐T‐DOX exerted comparable endocytosis to that of T‐DOX and was significantly weaker than that of free DOX (**Figure** **3**A and Figure S8, Supporting Information). This occurred mainly because the superficial cilengitide of MC‐T‐DOX even could be decreased to a lower density (<0.02 mol%) through MT1‐MMP cleavage. Visually, confocal images also revealed that MC‐T‐DOX displayed markedly weaker red fluorescence (DOX) within the nuclear areas than free DOX, confirming its limited endocytosis by ECs (Figure 3B).

Next, these formulations were incubated with HUVECs to examine their cytotoxicity by MTT assay (Figure 3C). The liposomal DOX from T‐DOX, MC‐T‐DOX, and NMC‐T‐DOX induced significantly negligible cytotoxicity toward HUVECs compared to that of free DOX. Especially, for MC‐T‐DOX, we observed 83% cell viability even at a DOX concentration of 10 µg mL^−1^. This may be attributed to poor cellular uptake of these liposomal DOX. Taken together, these results suggest that MC‐T‐DOX is relatively innocuous to ECs.

Considering the good safety of MC‐T‐DOX, we moved to investigate if MC‐T‐DOX produced MT1‐MMP‐activated proangiogenic effect using in vitro model systems of angiogenesis. Nanomolar cilengitide has been demonstrated to promote tumor angiogenesis by promoting EC migration.^[^ ^11^, ^12^ ^]^ Hence, Scratch wound assay was chosen because it closely recapitulates the processes of EC migration observed in vivo. For this, HUVECs were incubated with different formulations (MC‐T, cilengitide, T‐DOX, MC‐T‐DOX, NMC‐T‐DOX) at a cilengitide concentration of 2 nm for 24 h and migration area was calculated. Migration area in T‐DOX group was not significantly different compared with the DMEM‐treated group, indicating no promoting effect on HUVEC migration from the cilengitide‐free nanovesicle itself. Migration area in NMC‐T‐DOX‐treated group was only comparable to that of the DMEM‐treated group, indicating a negligible effect (Figure 3D,E). The formation of focal contacts is necessary for cell migration, which requires higher RGD surface densities.^[^ ^19^ ^]^ Thus, this result suggests that the low density of cilengitide was not enough to trigger formation of focal contacts for endothelial migration. In contrast, MC‐T‐DOX resulted in significantly higher migration area than the migration area observed in the DMEM‐treated group (*p* < 0.01) and comparable to that of the cells treated with cilengitide, indicating a promoting effect on HUVEC migration (Figure 3D,E). Unlike modified cilengitide, free cilengitide is not limited to the surface density and free to cluster integrin for forming focal contacts. Hence, the promoting effect of MC‐T‐DOX on HUVEC migration may be owing to its activation by MT1‐MMP to release free cilengitide. In addition, comparable migration area was observed between MC‐T‐DOX and MC‐T group, confirming that MC‐T‐DOX is safe to HUVECs.

Next, to investigate the involvement of cilengitide concentration in the promoting migration ability of MC‐T‐DOX, we incubated cilengitide or MC‐T with HUVECs and measured the migration area, as cilengitide concentration ranged from 2 nm to 2 µm. As indicated, cilengitide and MC‐T displayed a comparable promoting effect on HUVEC migration only as cilengitide concentration was less than 200 nm (Figure S9, Supporting Information). At the concentration of 2 µm, in cilengitide or MC‐T‐treated group, HUVECs greatly shrank and detached to enlarge wound area, in particular detached large cell clusters were observed in cilengitide‐treated group, precluding analysis of migration area (Figure 3F). This result occurred because micromolar concentrations of cilengitide completely blocked the interaction of *α*v*β*3 with its extracellular matrix ligands, which is required for the survival of these ECs.^[^ ^14^, ^15^ ^]^ Consistent with the results of previous studies, these findings confirmed that MC‐T promoted angiogenesis only at a nanomolar concentration of cilengitide, but exerted antiangiogenic effect at a micromolar concentration.^[^ ^12^ ^]^

To further examine the MT1‐MMP‐activated proangiogenic ability of MC‐T‐DOX, we chose a more complete in vitro model of tube formation assays, which encompasses all the steps involved in the angiogenic process: adhesion, migration, protease activity, alignment and tube formation observed in vivo.^[^ ^28^ ^]^ For this, HUVECs were incubated with different formulations (MC‐T, cilengitide, T‐DOX, MC‐T‐DOX, NMC‐T‐DOX) at a cilengitide concentration of 2 nm for 8 h, then tube formation was imaged and analyzed. Consistent with the results of the scratch wound assays, the junction number and tube length in T‐DOX or NMC‐T‐DOX group were only comparable to those in DMEM‐treated group, indicating no promoting effect on tube formation. In contrast, the junction number and tube length of the MC‐T‐DOX‐treated group were significantly greater than those of the DMEM‐treated group and comparable to those of the cells treated with cilengitide or MC‐T, indicating a promoting effect on tube formation (Figure 3G,H). These results suggest that after activated by MT1‐MMP to release free cilengitide, MC‐T‐DOX could promote EC migration, which ultimately resulted in improved tube formation in a more complete in vitro model system of angiogenesis.

To further explore if MC‐T‐DOX exhibited MT1‐MMP‐activated proangiogenic effect in vivo, we performed Matrigel plug assays. The Matrigel microenvironment contains a variety of extracellular matrix proteins and an angiogenic stimulus VEGF, and thereby replicates, at least in part, the tumor microenvironment; ECs can migrate into the plugs in a similar manner as they can into tumors, forming capillary networks and microvessels.^[^ ^29^ ^]^ Numerous studies have used implanted Matrigel plugs to evaluate the efficacy of tumor‐associated angiogenesis modulating agents in vivo,^[^ ^30^ ^]^ when treatment agent pharmacokinetics is considered. Especially, these angiogenic ECs highly express MT1‐MMP.^[^ ^20^, ^21^ ^]^ These facts support the applicability of this model for evaluating MT1‐MMP‐activated proangiogenic ability of MC‐T‐DOX in vivo.

The ECs that line the interior of blood vessels are directly exposed to blood circulation. The approximate blood volume of a mouse is 50–70 mL kg^−1^. When MC‐T‐DOX was administered at a DOX dose of 3 mg kg^−1^, which corresponded to a low dose of the modified cilengitide (8.5 nm kg^−1^). Even if MC‐T‐DOX only distributed in blood postinjection, the exposure concentration of MC was less than 200 nm (120–170 nm). This implied that tumor ECs would be exposed to a “proangiogenic” (nanomolar) concentration of cilengitide as indicated by in vitro assays. We hence chose this dose to investigate MT1‐MMP‐activated proangiogenic effect of MC‐T‐DOX in vivo.

For this, male nude mice were subcutaneously implanted with VEGF containing Matrigel. Then, these animals were intravenously injected with PBS, free cilengitide, T‐DOX, MC‐T‐DOX, or NMC‐T‐DOX at an equal DOX dose of 3 mg kg^−1^ and cilengitide dose of 8.5 nm kg^−1^ every 4 days for three cycles. On day 12, these plugs were removed and imaged. The PBS‐, T‐DOX‐, or NMC‐T‐DOX‐treated plugs appeared pale and avascular, and the cilengitide‐treated plugs were light red with marginal angiogenesis. In contrast, the MC‐T‐DOX‐treated plugs were much darker red, with visible vessels (Figure 3I). Then, anti‐CD31 immunostaining was used to examine the population of ECs within the Matrigel plugs: CD31‐negative cells within the plugs might be pericytes or stromal cells recruited during angiogenesis (Figure 3J).^[^ ^29^ ^]^ Consistent with this visual inspection, EC abundance in T‐DOX or NMC‐T‐DOX group was comparable to that in PBS‐treated plugs, indicating no proangiogenic effect. These results coincided well with in vitro results, which resulted from the inability of these cilengitide‐free or MT1‐MMP‐insensitive nanovesicles to trigger EC migration for angiogenesis. In contrast, EC relative abundance in MC‐T‐DOX‐treated plugs was significantly higher by 4.6‐fold than that in the PBS‐treated plugs (*p* < 0.001; Figure 3K), indicating a significantly improved proangiogenic effect. This result was in agreement with in vitro results and may be attributed to activation of MC‐T‐DOX by MT1‐MMP on angiogenic ECs within plugs to release cilengitide, which accordingly promoted EC migration and angiogenesis. In addition, EC abundance in cilengitide‐treated plugs was slightly higher than that in the PBS‐treated plugs and considerably lower than that in MC‐T‐DOX‐treated plugs (Figure 3J,K), which was different from the in vitro results and may be attributed to its rapid elimination from in vivo.^[^ ^16^ ^]^ Taken together, these in vivo results supported our idea that MC‐T‐DOX not only prolonged cilengitide circulation time, but also achieved effective cilengitide activation by responding to MT1‐MMP on tumor ECs, thereby exhibiting the superior proangiogenic effect in vivo.

### 2.4 Effect of MC‐T‐DOX on Tumor Vascular Perfusion

**Figure 4:**
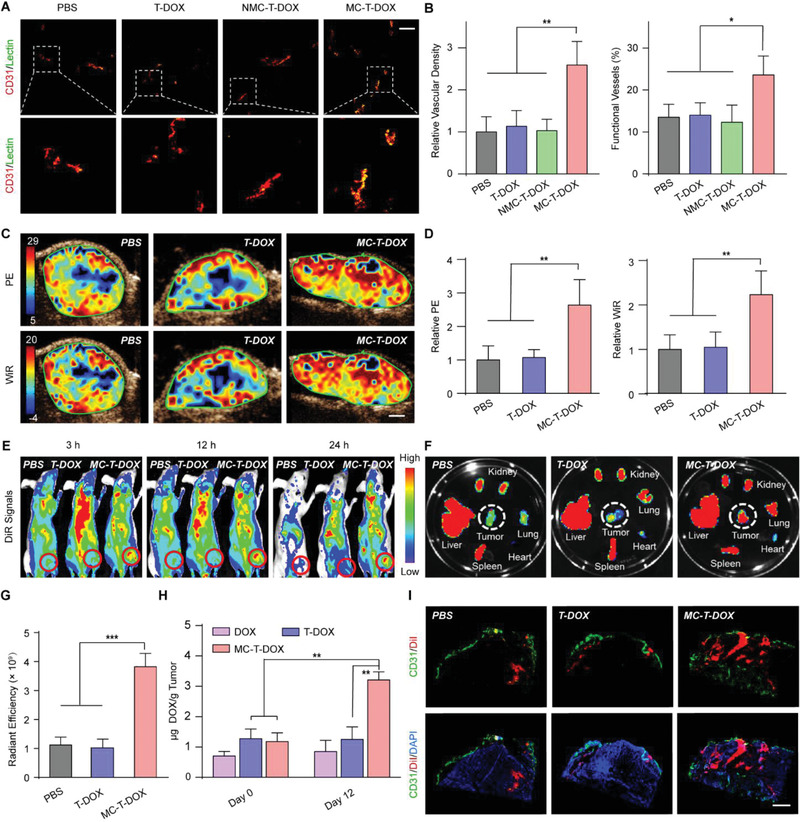
Effect of MC‐T‐DOX treatment on tumor vascular perfusion and tumor delivery of MC‐T‐DOX. BxPC‐3‐bearing mice were treated with the indicated formulations every 4 days at an identical DOX dose of 3 mg kg^−1^ and on day 12 tumor vascular perfusion and tumor delivery of MC‐T‐DOX were evaluated. A) Representative images of tumor blood vessels and functional blood vessels and B) quantitative analysis of blood density and percent of functional blood vessels. Scale bar: 100 µm. Tumor blood vessel density was determined by counting the number of CD31–positive blood vessels per 200 × field and then dividing by the corresponding area of the tumor (*n* = 7). C) Representative ultrasound images and D) statistical analyses of peak enhancement (PE) and wash‐in rate (WiR) (*n* = 5). Scale bar: 2 mm. The “relative” value refers to the ratio of the value in each group relative to that in the PBS group. E–G) BxPC‐3‐bearing mice were treated as described above, and on day 12, these animals were injected with DiR‐labeled TSLs. E) In vivo imaging of DiR‐labeled TSLs (The red circles show the position of the tumor xenografts.), F) ex vivo imaging of DiR‐labeled TSLs (The white circles show the tumor tissues.) and G) semiquantitative fluorescence intensities of the excised tumors at 24 h postinjection (*n* = 3). H) BxPC‐3‐bearing mice were treated with DOX, T‐DOX, or MC‐T‐DOX every 4 days. On day 0 and day 12, at 3 h postinjection, the intratumoral DOX amount was measured (*n* = 3). I) BxPC‐3‐bearing mice were treated as described above. On day 12, these animals were injected with DiI‐labeled TSLs and intratumoral distribution of DiI‐labeled TSLs was imaged at 24 h postinjection (blue: DAPI; green: CD31; red: DiI). Scale bar: 500 µm. Data are presented as mean ± SD. *p < 0.05, **p < 0.01, ***p < 0.001 among the marked groups using nonparametric two‐tailed analysis of variance.

Tumor ECs highly express MT1‐MMP.^[^ ^20^, ^21^ ^]^ Considering the promising MT1‐MMP‐activated proangiogenic effect of MC‐T‐DOX in in vivo Matrigel plugs, we further investigated whether it can accordingly improve tumor blood perfusion in extremely hypoperfused BxPC‐3 pancreatic cancer models.^[^ ^31^ ^]^ For this, BxPC‐3‐bearing mice were treated with PBS, T‐DOX, NMC‐T‐DOX, or MC‐T‐DOX at an identical DOX dose of 3 mg kg^−1^ every 4 days for three cycles. On day 12, the tumor vascular density and function were evaluated (**Figure** **4**A,B). Only low tumor vascular density and percentage of functional vessels were observed in T‐DOX or NMC‐T‐DOX group, which was comparable to those in PBS group, indicating negligible effect on tumor angiogenesis or vascular function. These results were consistent with the results of Matrigel plug assays and revealed that in pancreatic tumor microenvironment these cilengitide‐free or MT1‐MMP‐insensitive cilengitide containing nanovesicles had no proangiogenic ability. In contrast, the tumor vascular density (CD31^+^) of MC‐T‐DOX‐treated mice was significantly increased by 2.6‐fold (*p* < 0.01) compared with that of PBS‐treated mice, indicating significantly improved angiogenesis. Moreover, the lectin labeling results also showed that the percentage of functional vessels (CD31^+^ plus lectin) was significantly increased from 13.5% in the PBS group to 23.6% in the MC‐T‐DOX group (*p* < 0.05), indicating that these newly formed blood vessels had increased function (Figure 4B). Taken together, these results suggest that in pancreatic tumor microenvironment, MC‐T‐DOX could promote tumor angiogenesis and vascular function after activated by MT1‐MMP on tumor ECs to release free cilengitide.

To further determine if MC‐T‐DOX could effectively improve tumor blood perfusion, we performed quantitative contrast‐enhanced ultrasonography. Briefly, the BxPC‐3 bearing mice were treated with PBS, T‐DOX, or MC‐T‐DOX at an identical DOX dose of 3 mg kg^−1^ every 4 days for three cycles. Then, on day 12, these animals were subjected to quantitative contrast‐enhanced ultrasonography. Representative parametric images of peak enhancement (PE) and wash‐in rate (WiR) for the same animal are shown in Figure 4C, which are indicative of blood volume and blood flow rate, respectively.^[^ ^32^ ^]^ The blood volume and blood flow rate in MC‐T‐DOX‐treated xenografts, were both more than doubled relative to PBS‐treated xenografts (*p* < 0.01), while the blood volume and blood flow rate in T‐DOX‐treated xenografts was only comparable to that in PBS‐treated xenografts (Figure 4D). The significantly improved blood perfusion in MC‐T‐DOX‐treated xenografts over T‐DOX‐treated xenografts may be because MC‐T‐DOX treatment effectively promoted tumor angiogenesis and vascular function.

In addition, we noted that within the MC‐T‐DOX‐treated xenografts, contrast enhancement was more homogenous throughout the tumor mass than it was for tumors of PBS‐ or T‐DOX‐treated xenografts, wherein enhancement was mostly restricted to the tumor periphery (Figure 4C). This finding indicated that MC‐T‐DOX can recruit blood flow into previously unperfused tumor regions, thereby decreasing the intervascular interstitial distance. Collectively, these results revealed that in the extremely hypoperfused BxPC‐3 pancreatic cancer models, MC‐T‐DOX treatment effectively improved tumor blood perfusion, in particular by decreasing the intervascular interstitial distance, thereby providing a huge opportunity to improve tumor drug delivery.

### 2.5 Tumor Delivery of MC‐T‐DOX

Considering that MC‐T‐DOX could effectively improve tumor blood perfusion, we moved to investigate whether this effect resulted in enhanced tumor delivery of itself. To visually track in vivo delivery of MC‐T‐DOX, we prepared 1,10‐dioctadecyl‐3,3,30,30‐tetramethylindo‐tricarbocyanineiodide (DiR)‐ and 1,1′‐dioctadecyl‐3,3,3′,3′‐tetramethylindocarbocyanine perchlorate (DiI)‐labeled TSLs, which had a comparable size (≈100 nm, Table S1, Supporting Information) to that of MC‐T‐DOX. Then, BxPC‐3‐bearing mice were treated with PBS, T‐DOX, or MC‐T‐DOX every 4 days for three cycles as described above. On day 12, these animals were intravenously injected with DiR‐labeled TSLs and fluorescence signal was examined through fluorescence imaging. Among all the treatment groups, the strongest fluorescence signals were observed in the MC‐T‐DOX‐treated xenografts at all timepoints (Figure 4E). At 24 h postinjection, the animals were sacrificed, and major tissues and tumors were excised and imaged. Consistently, the strongest fluorescence signals appeared in MC‐T‐DOX‐treated tumor. Semiquantitative data further confirmed that the fluorescence intensity in MC‐T‐DOX‐treated xenografts was significantly increased compared with that in the PBS‐ or T‐DOX‐treated xenografts (*p* < 0.001) (Figure 4F,G). These results indicated that MC‐T‐DOX treatment could improve tumor accumulation of 100‐nm TSL nanovesicle, namely itself, through improving the tumor blood perfusion. Generally, nanovesicles selectively extravasate into the tumor tissue from the leaky tumor vessels through the enhanced penetration and retention (EPR) effect. Hence, these results also suggest that these newly formed functional vessels by MC‐T‐DOX treatment might be leaky, allowing extravasation of these 100‐nm nanovesicles into tumor.

Moreover, accumulation of DiR‐labeled TSLs in normal tissues such as hearts, livers, spleens, lungs and kidneys was not significantly different in MC‐T‐DOX‐treated mice compared with that in PBS‐ or T‐DOX‐treated mice (Figure S10, Supporting Information). These findings indicated that MC‐T‐DOX treatment had a negligible effect on blood perfusion in normal tissues, due to minimal expression of *α*v*β*3 on normal ECs.^[^ ^33^ ^]^ Chemotherapeutic drugs, such as DOX, have dose limiting toxicities. Hence, the specific modulation of tumor vascular promotion has the potential to be more effective and less harmful to normal tissues.

To further investigate effect of MC‐T‐DOX treatment on tumor accumulation of itself, we determined pharmacokinetics of MC‐T‐DOX and its tumor accumulation changes before and after its treatment. First, pharmacokinetics of MC‐T‐DOX was evaluated in tumor free Sprague Dawley rats (Figure S11 and Table S2, Supporting Information). The pharmacokinetics of liposomal DOX from MC‐T‐DOX and T‐DOX was prolonged compared to free DOX. For example, the clearance half time (*t*_1/2_) and the area under curve (AUC) of DOX in MC‐T‐DOX were increased by 5.0‐ and 18.2‐fold than that in free DOX, respectively, indicating a long‐circulating effect (Table S2, Supporting Information). Indeed, MC‐T‐DOX displayed a comparable pharmacokinetic profile to T‐DOX, indicating that low‐density MC incorporation had a negligible effect on the pharmacokinetics of MC‐T‐DOX (Figure S11, Supporting Information).

Then, BxPC‐3‐bearing mice were intravenously injected with DOX, T‐DOX, or MC‐T‐DOX at a DOX dose of 3 mg kg^−1^ every 4 days. On day 0 and day 12, the DOX amount in the tumors was measured (Figure 4H). On day 0 when MC‐T‐DOX had no enough time to improve tumor blood supply, the intratumoral DOX amount in MC‐T‐DOX was comparable to that in T‐DOX, which was consistent with their comparable blood pharmacokinetics. DOX displayed the lowest tumor accumulation due to its rapid elimination. In DOX or T‐DOX‐treated mice, intratumoral DOX concentration on day 12 was still comparable to that on day 0, because they could not increase tumor blood perfusion. In contrast, in MC‐T‐DOX‐treated mice, intratumoral DOX amount on day 12 was significantly increased by 2.7‐fold relative to that on day 0 (*p* < 0.01) because its treatment could significantly improve tumor blood perfusion. Collectively, these data suggest that tumor accumulation of MC‐T‐DOX was significantly improved after its treatment.

Next, we further investigated the effect of MC‐T‐DOX treatment on tumor distribution of itself. Briefly, BxPC‐3‐bearing mice were treated as described above and on day 12 DiI‐labeled TSLs were injected into these animals. Then, at 24 h postinjection, tumor distribution of DiI‐labeled TSLs was checked. In the PBS‐ or T‐DOX‐treated mice, scarce tumor blood vessels were mostly located on the tumor periphery, which accordingly resulted in limited distribution of DiI‐labeled nanovesicles into tumor. In contrast, in the MC‐T‐DOX‐treated mice, rich tumor blood vessels were well‐distributed, which accordingly delivered DiI‐labeled TSLs more widely into the tumor (Figure 4I). These results suggest that MC‐T‐DOX treatment could effectively promote tumor distribution of itself through improving tumor blood perfusion, in particular by decreasing intervascular interstitial distance. Then, these encapsulated DOX in MC‐T‐DOX became more accessible to tumor cells, which only need to traverse a shorter intravascular interstitial distance to reach tumor cells.

### 2.6 In Vivo Antitumor Effect of MC‐T‐DOX

Heat is reported to increase local blood flow. Before performing the antitumor studies, we first examined heat effect on tumor accumulation of MC‐T‐DOX. For this, we established bilateral BxPC‐3 tumor‐bearing mouse model and injected various formulations (DOX, T‐DOX, MC‐T‐DOX). At 2 h postinjection, the right‐side xenografts were subjected to hyperthermia (42 °C) for 1 h in a heated water bath while the left‐side xenografts were not heated. At 3 h postinjection, DOX amount in tumor was determined. As indicated, for these formulations, DOX accumulation within the heated xenografts was only slightly higher than that within the unheated xenografts, indicating an unimportant effect of heat treatment (Figure S12, Supporting Information). This result may be because DOX plasma concentration for these formulations had greatly declined during heat treatment, which was not enough to build up significant increase in tumor DOX accumulation despite the increased blood flow.

**Figure 5:**
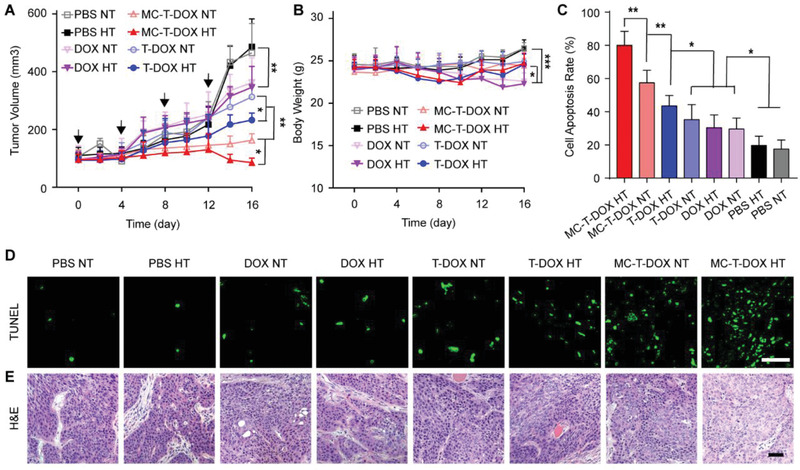
BxPC‐3‐bearing mice were intravenously treated with the indicated formulations every 4 days for four cycles. For the hyperthermia (HT) group, at 2 h postinjection, the xenografts were subjected to mild hyperthermia (42 °C) in a heated water bath for 1 h, while for the normothermia (NT) group, the xenografts were not heated. A) Tumor growth during the experiment, the arrows indicate the timepoints for treatment (*n* = 6). B) Body weight changes during the experiment (*n* = 6). C) Quantitative analysis of cell apoptosis (*n* = 6). D) TUNEL and E) H&E staining of the tumors performed at the end of the antitumor study. Scale bar: 50 µm. Data are presented as mean ± SD. *p < 0.05, **p < 0.01, ***p < 0.001 among the marked groups using nonparametric two‐tailed analysis of variance.

Encouraged by the superior capability of MC‐T‐DOX to improve tumor drug delivery, we then evaluated its antitumor activity in mice bearing hypoperfused BxPC‐3 tumors.^[^ ^31^ ^]^ When the tumor volume reached ≈100 mm^3^, the BxPC‐3‐bearing mice were divided into eight groups and intravenously administered PBS, DOX, T‐DOX, or MC‐T‐DOX with hyperthermia or normothermia. For the hyperthermia groups, at 2 h postinjection, the xenografts were subjected to hyperthermia (42 °C) for 1 h in a heated water bath. For the normothermia group, the xenografts were not heated. The treatments were repeated four times at a time interval of 4 days. The tumor volume and body weight were monitored during the treatment (**Figure** **5**A,B). Remarkably, among all the nonheated or heated treatment procedures, the MC‐T‐DOX group displayed the strongest therapeutic effect. In contrast, T‐DOX only moderately inhibited tumor growth (Figure 5A). This significantly improved therapeutic effect of MC‐T‐DOX over T‐DOX might be attributed to its significantly increased accumulation and distribution into tumors. DOX treatment produced the weakest therapeutic effect, due to its poor tumor accumulation. The PBS‐treated control mice displayed rapid tumor growth that was unaffected by heat treatment, indicating that heat treatment alone produced no therapeutic effect.

Heat treatment had an unimportant effect on tumor accumulation of DOX, T‐DOX, or MC‐T‐DOX as described above. However, we noted that heat treatment significantly improved tumor growth inhibition in the T‐DOX‐ or MC‐T‐DOX‐treated mice relative to a negligible effect on tumor growth in the DOX‐treated mice. In particular, MC‐T‐DOX plus hyperthermia almost induced complete tumor regression (Figure 5A). Hence, the significantly improved therapeutic efficacy may be due to heat‐triggered DOX release from T‐DOX or MC‐T‐DOX, which significantly improved DOX penetration and cellular uptake in the interstitium. Collectively, MC‐T‐DOX with hyperthermia displayed significantly enhanced therapeutic efficacy, which could be ascribed to the improved accumulation and wider accumulation of MC‐T‐DOX into tumor benefiting from the MT1‐MMP‐mediated activation of cilengitide in the tumor vessels, and enhanced DOX bioavailability through heat‐triggered DOX release in the interstitium.

To uncover the mechanism that enabled the superior antitumor effect of MC‐T‐DOX, we then prepared and examined the tumor slices for TUNEL (Figure 5C,D) and hematoxylin‐eosin (H&E) staining (Figure 5E). Massive nuclei damage and cytosol degradation were found in the MC‐T‐DOX with hyperthermia group using H&E staining (Figure 5E), indicating notable apoptosis of the tumor cells. The TUNEL assay revealed that the percentage of apoptotic cells in the MC‐T‐DOX with hyperthermia group (80.0%) was significantly greater than that in the other treatment groups (Figure 5C).

In addition, the systemic toxicity of MC‐T‐DOX was evaluated by the body weight changes of the mice during the treatment and the H&E staining of major organs. Free DOX‐treated mice exhibited a significant decrease in body weight (Figure 5B) and typical myocardial injury by histological assay (Figure S13, Supporting Information), indicating systemic toxicity and acute cardiotoxicity. In contrast, no significant body weight loss or obvious histological damage in the major organs were observed in the MC‐T‐DOX or the other treatment groups, indicating good biosafety.

## 3 Conclusion

To achieve effective and specific vascular promotion for improving tumor drug delivery, we constructed a smart nanovesicle, MC‐T‐DOX. In the hypoperfused pancreatic cancer model, MC‐T‐DOX might be activated by MT1‐MMP on tumor ECs to release cilengitide; this locally released cilengitide improved tumor blood perfusion, thereby resulting in an improved accumulation and a wider distribution of MC‐T‐DOX into tumors. Then, in the interstitium, heat‐triggered DOX release from MC‐T‐DOX significantly improved DOX bioavailability. Due to the improved drug delivery, MC‐T‐DOX significantly inhibited tumor growth in the pancreatic cancer mouse model. Moreover, ECs in different types of tumors share a common feature‐high expression of *α*v*β*3^[^ ^33^, ^34^ ^]^ and MT1‐MMP^[^ ^20^, ^21^ ^]^; thus, MC‐T‐DOX might also be applied to other hypoperfused tumors such as breast cancer. Taken together, our strategy of combining the modulation of tumor vascular promotion with smart nanodrug delivery holds great potential to improve drug delivery and chemotherapeutic efficacy in a wide range of hypoperfused tumors. We anticipate that these findings will inspire the development of novel nanodrug therapy strategies for hypoperfused tumors in the near future.

## 4 Experimental Section

##### Preparation of MC‐T‐DOX

TSLs, consisting of DPPC/DSPC/DSPE‐PEG2000 in a molar ratio of 56:40:4 (see Materials section in the Supporting Information), were prepared using a thin‐film hydration method. Briefly, DPPC, DSPC, and DSPE‐PEG2000 were dissolved in chloroform and methanol (90:10, v/v) and evaporated at 30 °C to remove the organic solvents. The lipid film was hydrated with ammonium sulfate buffer (pH 5.5). The suspension was extruded at 50 °C using an Avanti mini extruder and subjected to gel filtration through Sepharose CL‐4B to establish an ammonium sulfate gradient. Afterward, DOX‐loaded TSLs (T‐DOX) were prepared by loading DOX into the TSLs at 39 °C with an initial DOX/lipid ratio of 0.15:1 (mol/mol) as described previously.^[^ ^35^ ^]^ For MC synthesis and its MT1‐MMP responsiveness assay, see the Supporting Information. MC‐T‐DOX was prepared via a postinsertion method by gently incubating the micellar solution of MC (at a ratio of 0.055 mol% with respect to phospholipid) with T‐DOX at 37 °C for 1 h.^[^ ^23^ ^]^ MC‐T was prepared by incubating MC with blank TSLs. In addition, DiR‐ or DiI‐labeled TSLs were also prepared using this thin‐film hydration method by adding DiR or DiI into the phospholipid solution. Unloaded DOX, MC, DiR, or DiI were removed by gel filtration through Sepharose CL‐4B.

##### Characterization of MC‐T‐DOX

The hydrodynamic diameter and zeta potential of the liposomes were determined using dynamic light scattering at 25 °C in a Nano ZS zetasizer (Marvern, UK). Morphological observation of T‐DOX, NMC‐T‐DOX, and MC‐T‐DOX was performed using a Cryo‐TEM (Tecnai 12 electron microscope, USA) at an accelerating voltage of 200 KV. DOX was released from the TSLs using methanol and measured via HPLC. MC‐T‐DOX was ultracentrifuged at 300 000 g for 30 min, then MC was released by dissolving the MC‐T‐DOX precipitate in Triton X‐100 (10%) and MC content was determined via HPLC. The encapsulation efficiency (EE) and drug loading efficiency (DLE) of DOX and MC were calculated as indicated in Equations (1) and (2), respectively. Their values are reported as the mean ± SD (*n* = 3).

(1) $$\begin{array}{c} \mathrm{EE}\;\left(\%\right)\;=\;\frac{\mathrm{weight}\;\mathrm{of}\;\mathrm{the}\;\mathrm{drugs}\;\mathrm{in}\;\mathrm{liposomes}}{\mathrm{weight}\;\mathrm{of}\;\mathrm{the}\;\mathrm{feeding}\;\mathrm{drugs}} \end{array}$$

(2) $$\begin{array}{c} \mathrm{DLE}\;\left(\%\right)\;=\;\frac{\mathrm{weight}\;\mathrm{of}\;\mathrm{the}\;\mathrm{drugs}\;\mathrm{in}\;\mathrm{liposomes}}{\mathrm{weight}\;\mathrm{of}\;\mathrm{the}\;\mathrm{liposomes}\;\mathrm{and}\;\mathrm{drugs}} \end{array}$$

For MT1‐MMP responsiveness assay of MC‐T, see Supporting Information.

##### Bioavailability of MC‐T‐DOX in Tumor Spheroids

For cellular uptake and cytotoxicity of MC‐T‐DOX toward BxPC‐3, see Supporting Information. BxPC‐3 tumor spheroids were generated using a liquid overlay method as described previously.^[^ ^36^ ^]^ Briefly, 48‐cell plates were covered with 150 µL of hot agarose solution (2% w/v) per cell and cooled to room temperature. BxPC‐3 cells were seeded into each cell at a density of 7500 cells per well and incubated at 37 °C to form spheroids. When the spheroid diameter reached ≈400 µm, DOX, T‐DOX, or MC‐T‐DOX, which had been subjected to pretreatment with mild hyperthermia (42 °C) or normothermia (37 °C) in a water bath for 1 h, were added to the tissue culture plate at a final DOX concentration of 10 µg mL^−1^. After 1 h of incubation at 37 °C, the spheroids were washed with PBS to remove any formulations, fixed with 4% formaldehyde, and observed under a CLSM (FV1000, Olympus, Japan). To determine the cellular uptake of DOX within the spheroid cells, postincubation as described above, the spheroids were collected, washed, and trypsinized into individual cells. Afterward, the cells were washed with PBS and lyzed with DMSO. The supernatants were collected after centrifugation to obtain cell lysates. Then, the DOX content was measured with a microplate reader, and the protein content was determined using a BCA assay. Cellular uptake of DOX for different treatments is expressed as normalized DOX amounts to protein amounts.

##### Scratch Wound Assay

For cellular uptake and cytotoxicity assays of MC‐T‐DOX toward HUVECs, see Supporting Information. HUVECs (see the Cell Lines section in Supporting Information) were seeded in 6‐well plates at a density of 3 × 10^5^ cells per well and allowed to adhere overnight. The next day, after cells were serum‐deprived for 2 h, the confluent monolayer was scratched with a pipette tip and washed with PBS to eliminate free cells. The scratches at time zero were imaged. Then, HUVECs were incubated with serum‐free DMEM medium containing 50 ng mL^−1^ VEGF and supplemented with cilengitide, MC‐T, T‐DOX, MC‐T‐DOX, NMC‐T‐DOX at an identical cilengitide concentration of 2 nm. Herein, VEGF was used as an angiogenic stimulus. After 24 h of incubation at 37 °C, the scratches were photographed again. To further investigate the involvement of cilengitide concentration, the effect of cilengitide or MC‐T on HUVEC migration was further measured using the abovementioned method, as cilengitide concentrations ranged from 20 nm to 2 µm. ImageJ software was used to quantify the area of the scratch wound before and after migration. The migration area was calculated using Equation (3).^[^ ^12^ ^]^

(3) $$\begin{array}{ccc} \mathrm{Migration}\;\mathrm{area} & = & \left(\mathrm{area}\;\mathrm{of}\;\mathrm{scratch}\;\mathrm{wound}\;\mathrm{at}\;\mathrm{time}\;\mathrm{zero}\right)\\ & & -\;\left(\mathrm{area}\;\mathrm{of}\;\mathrm{scratch}\;\mathrm{wound}\;\mathrm{at}\;\mathrm{time}\;t\right) \end{array}$$

##### Tube Formation Assay

Matrigel was thawed overnight on ice at 4 °C, and then added to precooled 96‐well plates, followed by incubation at 37 °C to solidify. Diluted HUVECs in 100 µL of medium containing 50 ng mL^−1^ VEGF supplemented with cilengitide, MC‐T, T‐DOX, MC‐T‐DOX, or NMC‐T‐DOX at an identical cilengitide concentration of 2 nm were added to the plates on the layer of Matrigel at 1.5 × 10^4^ cells per well. Here, VEGF was used as an angiogenic stimulus. After 8 h of incubation, the tubes formed per well were photographed using an inverted microscope (DMI 4000B, Leica, Germany). ImageJ software was used to calculate the number of junctions and length of tubes.^[^ ^37^ ^]^

##### Matrigel Plug Assay

All animal procedures were carried out under the guidelines approved by the Institutional Animal Care and Use Committee of the Shanghai Institute of Material Medica, Chinese Academy of Sciences (IACUC No. 2017‐12‐GY‐34 and 2018‐05‐GY‐41 for nude mice and rats, respectively). Fifteen male Balb/c nude mice (20 ± 2 g) (see Animals section in Supporting Information) were randomly divided into five groups (*n* = 3) and subcutaneously injected with 300 µL of VEGF containing Matrigel in the ventral surface of the groin area. Each 300 µL plug contained 200 ng of VEGF as an angiogenesis stimulus. The mice were then intravenously injected with PBS, cilengitide, T‐DOX, NMC‐T‐DOX, or MC‐T‐DOX at an equal cilengitide dose of 8.5 nm kg^−1^ and DOX dose of 3 mg kg^−1^ every 4 days for three cycles. On day 12, the mice were sacrificed, and the plugs were removed and photographed. Afterward, the plug samples were snap frozen for cryosectioning. Then, these frozen sections were further stained using anti‐CD31 antibody to label ECs and imaged under a CLSM.

##### Measurement of Functional and Whole Vessels in Tumor

A subcutaneous tumor‐bearing nude mouse model was established by inoculating 8 × 10^6^ BxPC‐3 cells into the subcutaneous tissue of the right hind limbs of nude mice. When the tumor volume reached ≈100 mm^3^, the mice were randomly divided into four groups and intravenously injected with PBS, T‐DOX, NMC‐T‐DOX, or MC‐T‐DOX at an identical cilengitide dose of 8.5 nm kg^−1^ and DOX dose of 3 mg kg^−1^ every 4 days for three cycles. On day 12, the animals were injected with *Bandeiraea simplicifolia* lectin (0.1 mg kg^−1^). At 20 min postinjection, the animals were sacrificed. The tumors were removed and processed for sections and immunofluorescence staining with anti‐CD31 antibody to label whole tumor vascular ECs. The slides were observed for total tumor vessels and functional vessels under CLSM. Tumor blood vessel density was calculated by counting the number of CD31—positive blood vessels per 200 × field and then dividing by the corresponding area of tumor. The percentages of lectin and CD31 dual‐positive functional vessels were estimated using ImageJ software.

##### Tumor Perfusion Assay

The mice bearing BxPC‐3 tumors were treated with PBS, T‐DOX, or MC‐T‐DOX every 4 days for three cycles as described above. On day 12, contrast‐enhanced ultrasound imaging was carried out under isofluorane anesthesia using a Vevo LAZR imaging system (VisualSonics Inc., Toronto, Canada) according to the manufacturer's instructions. Briefly, the mice were injected with 100 µL of microbubble contrast agent via a tail vein catheter. Videos and images were acquired before, during, and after injection. The ultrasound images were processed and analyzed to calculate the relevant parameters, including peak enhancement (PE) and wash‐in rate (WiR), using VevoCQ contrast quantification software.

##### Tumor Accumulation of DiR‐Labeled TSLs After MC‐T‐DOX Treatment

The mice bearing BxPC‐3 tumors were treated with PBS, T‐DOX, or MC‐T‐DOX every 4 days for three cycles as described above. On day 12, the mice were injected with DiR‐labeled TSLs and imaged at the preset timepoints via an in vivo IVIS spectrum imaging system (PerkinElmer, USA). At 24 h postinjection, the mice were euthanized, and tumors and major organs were harvested for fluorescent imaging.

##### Tumor Accumulation of MC‐T‐DOX Before and After Treatment

The mice bearing BxPC‐3 tumors were intravenously injected with DOX, T‐DOX, or MC‐T‐DOX every 4 days. On day 0 and day 12, at 3 h postinjection, the animals were sacrificed, and tumors were removed. Then, the DOX amount in tumors was quantitatively analyzed. Briefly, tissue samples were weighed and homogenized using an ultrasonic cell smash (Ningbo Scientz Biotechnology) in acidified isopropanol containing 0.5% Triton X‐100. Then, the DOX amount in the supernatant was measured using a microplate reader, calculated and expressed as µg DOX per gram tumor.

##### Tumor Distribution of DiI‐Labeled TSLs After MC‐T‐DOX Treatment

The mice bearing BxPC‐3 tumors were treated with PBS, T‐DOX, or MC‐T‐DOX every 4 days for three cycles as described above. On day 12, the mice were injected with DiI‐labeled TSLs. At 24 h postinjection, the animals were sacrificed, and the tumors were removed and processed for sections and immunofluorescence staining with anti‐CD31 antibody to label vessels. The distribution of DiI‐labeled TSLs was observed under a microscope (DM6B, Leica, Germany).

##### In Vivo Antitumor Effect

Subcutaneous BxPC‐3 tumors in the right hind limbs were established as described above, and the treatment commenced when the tumor volume reached ≈100 mm^3^. The tumor‐burdened mice were divided into eight groups and intravenously injected with PBS, DOX, T‐DOX, or MC‐T‐DOX with hyperthermia or normothermia at an identical DOX dose of 3 mg kg^−1^ and a cilengitide dose of 8.5 nm kg^−1^. For the hyperthermia groups, at 2 h postinjection, the xenografts were subjected to mild hyperthermia (42 °C) in a heated water bath for 1 h according to the method previously described with minor modifications.^[^ ^26^, ^36^ ^]^ Briefly, the tumor‐bearing mice were anesthetized and fixed onto specially designed holders to ensure a steady position, where their tumors completely submerged in the water bath during the hyperthermia treatment. The water bath temperature was set to 43 °C, which had previously been calibrated to give a tumor temperature of 42 °C.^[^ ^36^ ^]^ For the normothermia group, the xenografts were not heated. The treatments were administered every 4 days for four cycles. The tumor diameter and body weight were measured every other day, and the tumor volume (*V*) was calculated with the equation: *V* = 1/2*ab* ^2^, where *a* and *b* indicate the long and short diameters of the tumor, respectively. At the end of the treatment, the animals were sacrificed, and the tumor and other major organs were removed. The major organs of hearts, livers, spleens, lungs, and kidneys were stained using H&E and photographed to evaluate the safety of various formulations. Furthermore, the tumor tissues were stained using H&E and TUNEL to evaluate the apoptosis levels.

##### Statistical Analysis

The results are expressed as mean ± SD. The differences between three or more independent groups were tested by nonparametric two‐tailed analysis of variance (ANOVA) followed by a Tukey post‐hoc test. In all cases, *p*‐value ≤ 0.05 was considered as being statistically significant. Statistical analysis was carried out using GraphPad Prism 7 Software.

## Conflict of Interest

The authors declare no conflict of interest.

## Supporting information

Supporting Information
