# Supplementary material for: MT1‐MMP‐Activated Liposomes to Improve Tumor Blood Perfusion and Drug Delivery for Enhanced Pancreatic Cancer Therapy
Source: Adv Sci (Weinh). 2020 Jul 10;7(17):1902746. doi: 10.1002/advs.201902746 (PMC7507343; doi:10.1002/advs.201902746)
Supplement: Supplementary file 1 — Supporting Information [file ADVS-7-1902746-s001.pdf]

# Supporting Information

## MT1-MMP-Activated Liposomes to Improve Tumor Blood Perfusion and Drug Delivery for Enhanced Pancreatic Cancer Therapy

*Yan Wei, Sha Song, Nianxiu Duan, Feng Wang, Yuxi Wang, Yiwei Yang, Chengyuan Peng, Junjun Li, Di Nie, Xinxin Zhang, Shiyan Guo, Chunliu Zhu, Miaorong Yu, Yong Gan*[*]

**Materials.** Cilengitide was purchased from Nanjing Leon Biological Technology Co., Ltd. (Nanjing, China). The phospholipids of 1,2-distearoyl-sn-glycero-3-phosphocholine (DSPC), 1,2-dipalmityol-sn-glycero-3-phosphocholine (DPPC), and 1,2-distearoyl-sn-glycero-3-phosphoethanolamine-N-PEG2000 (DSPE-PEG2000) were purchased from Shanghai Advanced Vehicle Technology Pharmaceutical L.T.D. Co (AVT) (Shanghai, China). Sulphur Hexafluoride Microbubbles for Injection was from Bracco Imaging B.V. (Switzerland). Growth factor-reduced Matrigel matrix was purchased from BD Biosciences (San Diego, California, USA). VEGF was purchased from Abbkine Scientific Co., Ltd. (California, USA). Human MT1-MMP recombinant protein was purchased from Thermo Fisher Scientific Inc. (Invitrogen, California, USA). CD31 goat polyclonal antibody was purchased from Wuhan Google Biological Technology Co. Ltd. (Wuhan, China). FITC conjugated lectin from *Bandeiraea simplicifolia* and 4′,6-diamidino-2-phenylindole dihydrochloride (DAPI) were from Sigma-Aldrich (St. Louis, MO). A rabbit MT1-MMP mAb was purchased from Abcam (Hong Kong). A mouse β-actin mAb, horseradish peroxidase (HRP)–conjugated goat anti-rabbit and goat anti-mouse antibody were purchased from Beyotime Biotechnology (Shanghai, China). RPMI 1640 medium, Dulbecco's Modified Eagle's medium (DMEM), fetal bovine serum, penicillin/streptomycin, and trypsin−EDTA solution (0.25%, trypsin with 0.53 mM EDTA) were purchased from Life Technologies Co. (Grand Island, NY, USA). All other chemicals and reagents were purchased from Sinopharm Chemical Reagent Co., Ltd. (Shanghai, China) if not mentioned otherwise.

**Cell Lines.** Human pancreatic cancer cell line BxPC-3 and human umbilical vein endothelial cell line HUVECs were purchased from the Cell Bank of the Chinese Academy of Sciences. BxPC-3 was maintained in RPMI 1640 medium, and HUVECs were cultured in DMEM medium. These media were supplemented with 10% fetal bovine serum, 100 U $mL^{-1}$ penicillin, and 100 μg $mL^{-1}$

streptomycin. All the cells were cultured at 37 °C in a 95% humidified atmosphere containing 5% $CO_2$.

**Animals.** Male Sprague Dawley rats (200 ± 20 g) and male BALB/c nude mice (20 ± 2 g) were ordered from Shanghai Sippr-BK Laboratory Animal Co., Ltd.

**MC and NMC Synthesis.** MT1-MMP-activated cilengitide (MC) was synthesized according to the standard Fmoc chemistry protocols (Scheme S1). The amino acids of MT1-MMP substrate dodecapeptide (KRRQLGLPALSβAla) were sequentially conjugated to the resin. Then, after Fmoc protection was removed using 25% piperidine in DMF, the substrate peptide was linked to cilengitide through a condensation reaction. Afterwards, after Dbe protection was removed by hydrazine hydrate, this peptide fragment was further condensed with stearic acid (C18 chain). Finally, MC was cleaved from the resin using TFA and purified using semipreparative reverse-phase HPLC. The successful synthesis of MC was confirmed using HPLC-UV/MS.

NMC was also synthesized following the same procedure, except that the MT1-MMP-sensitive linker of KRRQLGLPALSβAla was replaced with an MT1-MMP-insensitive linker of krrqlglpalsβala. The successful synthesis of NMC was confirmed using HPLC-UV/MS.

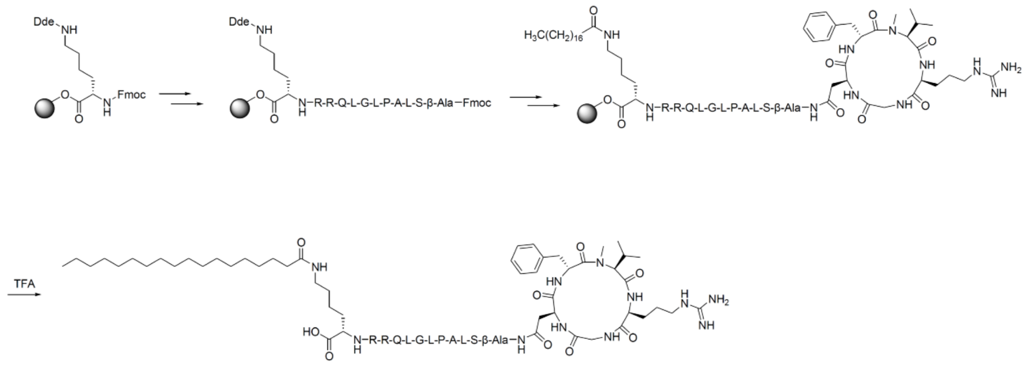


**Scheme S1.** Synthetic procedures of MC.

**MT1-MMP Responsiveness of MC.** MC was dissolved in MT1-MMP hydrolysis buffer (50 mM Tris, 10 mM $CaCl_2$, 150 mM NaCl, 0.05% Brij-35, pH 7.5, TCNB). Post-incubation at 37 °C

for 30 min, recombinant MT1-MMP (0.1 μg) in TCNB buffer with or without an MMP inhibitor GM6001 (100 μM) was added to the MC solution. The reaction lasted for 2 h at 37 °C. Afterwards, the reaction solution was rapidly transferred to ice-cold quenching reagent. The quenched samples were analyzed with HPLC-UV/MS as follows. In addition, MT1-MMP responsiveness of NMC was also evaluated using the abovementioned procedure.

**MT1-MMP Responsiveness of MC-T.** Concentrated solution of MC-T was incubated with recombinant MT1-MMP (0.1 μg) in TCNB for 2 h. Then, the mixture was ultracentrifuged at 300,000 g for 30 min to precipitate these TSLs. Afterwards, the supernatant was analyzed with HPLC-UV/MS to detect MT1-MMP cleavage products.

**HPLC-UV/MS Condition for MC Determination.** HPLC column, YMC-Pack ODS-AQ (250 × 4.6 mm, 5 μm); UV detector, 220 nm; flow rate, 0.8 mL $min^{-1}$; column temperature, 30 °C; gradient elution settings,

| T [min] | 0.1% formic acid in water | 0.1% formic acid in acetonitrile |
|---|---|---|
| 0 | 90% | 10% |
| 13.5 | 52% | 48% |
| 27 | 25% | 75% |
| 33.5 | 25% | 75% |
| 33.6 | 90% | 10% |
| 40 | 90% | 10% |

Triple-quadrupole tandem mass spectrometry with an electrospray ionization (ESI) interface was used. MS parameters: scan modes, positive ionization; capillary voltage, 3.5 kV; nebulizer, 35 PSI; gas temperature, 300 °C; gas flow, 10 L $min^{-1}$; sheath gas temperature, 350 °C; sheath gas flow, 10 L $min^{-1}$.

**Temperature-dependent DOX release.** For *in vitro* temperature-dependent DOX release, one

volume of liposomal DOX (T-DOX, MC-T-DOX or MC-T-DOX that had been subjected to MT1-MMP incubation for 2 h) in HEPES was added to nine volumes of preheated fetal bovine serum and incubated for 1 h under stirring at 37 °C or 42 °C in a thermal-shaker. DOX release at the preset timepoints was determined and calculated using a method described previously.[1]

**Cellular Uptake of MC-T-DOX by BxPC-3.** BxPC-3 were seeded into 12-cell plates at a density of 3 × $10^4$ cells/well and incubated to allow cell attachment. To investigate the involvement of heat treatment in cellular uptake, DOX, T-DOX, or MC-T-DOX were subjected to pretreatment of mild hyperthermia (42 °C) or normothermia (37 °C) for 1 h before use. Then, these formulations were incubated with BxPC-3 at an identical DOX concentration of 10 μg $mL^{-1}$ at 37 °C for 3 h, and lifted with dissociation buffer. DOX uptake was determined using a BD Bioscience FACSCalibur flow cytometer. Mean fluorescence intensity of each group was analyzed using FlowJo 7.6.1 software.

For confocal imaging, HUVECs were incubated with the indicated formulations at DOX concentration of 30 μg $mL^{-1}$ at 37 °C for 2 h. Afterwards, the cells were washed with cold PBS, mounted in Dako fluorescent mounting medium, and then observed under a confocal laser scanning microscope (CLSM) (FV1000, Olympus, Japan).

***In Vitro* Cytotoxicity Assay of MC-T-DOX toward BxPC-3.** BxPC-3 were seeded in 96-well plates at a density of 3000 cells/well and allowed to attach for 24 h. To investigate the involvement of heat treatment in cytotoxicity, DOX, T-DOX, or MC-T-DOX were subjected to pretreatment of mild hyperthermia (42 °C) or normothermia (37 °C) for 1 h before use. Then, these formulations were incubated with BxPC-3 for 24 h, as DOX concentration ranged from 0.001 to 10 μg $mL^{-1}$. Then, cell viability was analyzed using MTT assays.

**Cellular Uptake of MC-T-DOX by HUVECs.** HUVECs were seeded into 12-well plates at a density of 1 × $10^5$ cells/well and incubated for 24 h to allow cell adherence. Cells were then incubated with different formulations at an identical DOX concentration of 10 μg $mL^{-1}$ for 3 h, and

lifted with dissociation buffer. DOX uptake was determined using a BD Bioscience FACSCalibur flow cytometer. Mean fluorescence intensity of each group was analyzed using FlowJo 7.6.1 software.

For confocal imaging, HUVECs were incubated with DOX, T-DOX, NMC-T-DOX, or MC-T-DOX at an identical DOX concentration of 30 μg $mL^{-1}$ at 37 °C for 2 h. Then, the cells were washed using cold PBS, mounted in Dako fluorescent mounting medium, and then observed under CLSM.

***In Vitro* Cytotoxicity Assay of MC-T-DOX toward HUVECs.** HUVECs were seeded into 96-well plates at a density of 3000 cells/well and incubated for 24 h to allow cell attachment. Afterwards, the growth media were replaced with DOX, T-DOX, NMC-T-DOX or MC-T-DOX at DOX concentration of 0.001, 0.01, 0.1, 1 or 10 μg $mL^{-1}$ and incubated at 37 °C for 24 h. Then, cell viability was analyzed using MTT assays.

**Western blot assay.** The whole-cell lysate of HUVECs was prepared by ultrasonicating the cells in ice-cold cell lysis buffer. Afterwards, the whole proteins (30 μg) were resolved by 10% SDS-PAGE and electroblotted onto a polyvinylidene difluoride membrane. The blot was probed with a rabbit mAb to MT1-MMP (Abcam) and a mouse mAb to β-actin (Beyotime) followed by a horseradish peroxidase (HRP)–conjugated goat anti-rabbit (Beyotime) and goat anti-mouse antibody (Beyotime), respectively. Then, the proteins were detected using a Super ECL Detection Reagent (Yeasen) and visualized using a chemiluminescence imaging system (chemiScope 3300 min).

**Pharmacokinetics Study**. Sprague Dawley male rats (200 ± 20 g) were randomly divided into three groups (n = 3) and intravenously injected with DOX, T-DOX or MC-T-DOX at an identical cilengitide dose of 8.5 nM $kg^{-1}$ and DOX dose of 3 mg $kg^{-1}$, respectively. At preset timepoints, blood samples were drawn from the retinal vein plexus. The supernatant was collected after

centrifugation at 4000 rpm for 10 min and extracted using acidified isopropanol containing 0.5% Triton X-100 overnight at 4 °C. The pharmacokinetic profile was evaluated by measuring the blood DOX concentration using a microplate reader (Synergy H1, Biotek, USA).

**Effect of Heat on Tumor Accumulation of MC-T-DOX *In Vivo*.** Each Balb/c nude mouse was subcutaneously inoculated with BxPC-3 cells in the both hind limbs at $8 \times 10^6$ cells for each side. When the tumor volume reached ~300 $mm^3$, the mice were randomly divided into three groups ($n$ = 3) and intravenously injected with DOX, T-DOX, or MC-T-DOX at an identical DOX dose of 3 mg $kg^{-1}$. At 2 h post-injection, the right-side tumors were subjected to mild hyperthermia (42 °C) in a heated water bath for 1 h, while the left-side tumors were not heated as the control. Afterwards, the animals were sacrificed, and tumors were harvested. To determine DOX amounts, the tumor tissues were weighed and homogenized using an ultrasonic cell smash (Ningbo Scientz Biotechnology) in acidified isopropanol containing 0.5% Triton X-100. Then, DOX amounts in the supernatant was measured using a microplate reader, calculated and expressed as μg DOX/g tissue.

**Table S1.** Characterization of MC-T-DOX.

| Sample Name | Z-average [nm] | PDI | Zeta Potential [mV] | Encapsulation Efficiency [%] |
|---|---|---|---|---|
| T-DOX | 93.1 ± 3.5 | 0.140 ± 0.036 | -3.57 ± 0.30 | 87.2 ± 1.6 |
| MC-T-DOX | 100.2 ± 3.9 | 0.120 ± 0.020 | -2.17 ± 0.46 | 86.1 ± 2.2 |
| NMC-T-DOX | 101.3 ± 2.3 | 0.114 ± 0.016 | -2.24 ± 0.56 | 85.8 ± 2.4 |
| DiR-labeled TSL | 101.8 ± 4.1 | 0.123 ± 0.018 | -3.37 ± 1.40 | / |
| DiI-labeled TSL | 102.4 ± 3.8 | 0.132 ± 0.025 | -3.12 ± 0.90 | / |

Data are represented as mean ± SD (*n* = 3).

**Table S2.** Pharmacokinetics parameters of DOX, T-DOX and MC-T-DOX after intravenous injection *via* the tail vein in rats at an identical DOX dose of 3 mg $kg^{-1}$.

| Formulations | $t_{1/2}$ [h] | AUC (0-24 h)[a] [μg $mL^{-1}$ *h] | $C_{max}$[b] [μg $mL^{-1}$] | MRT (0-24 h)[c] [h] |
|---|---|---|---|---|
| DOX | 1.60 ± 0.23 | 4.04 ± 1.47 | 3.94 ± 1.04 | 1.34 ± 0.17 |
| T-DOX | 8.33 ± 4.26 | 72.83 ± 31.21 | 14.18 ± 2.77 | 7.56 ± 0.46 |
| MC-T-DOX | 8.05 ± 0.65 | 73.37 ± 29.92 | 14.49 ± 3.20 | 7.58 ± 0.53 |

Data are presented as mean ± SD (*n* = 3).

[a]Area under the curve (AUC).

[b]The maximum drug concentration ($C_{max}$).

[c]Mean residence time (MRT).

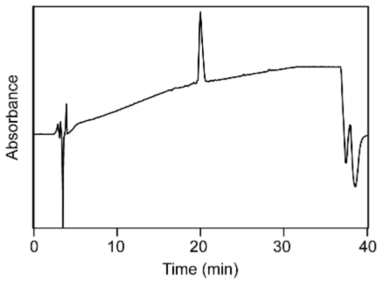


**Figure S1**. HPLC chromatogram of MC at 220 nm with the gradient elution.

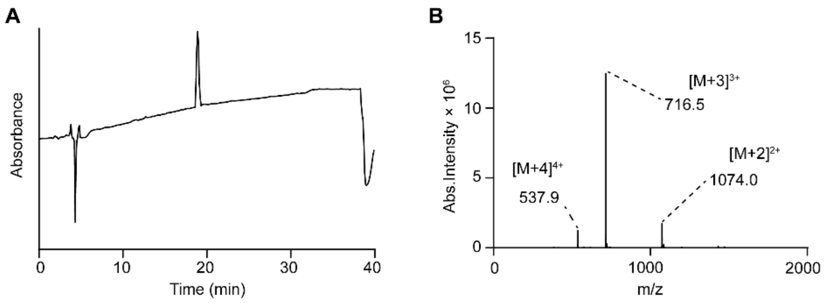


**Figure S2.** (A) HPLC chromatogram of NMC at 220 nm with the gradient elution. (B) MS spectrum of NMC.

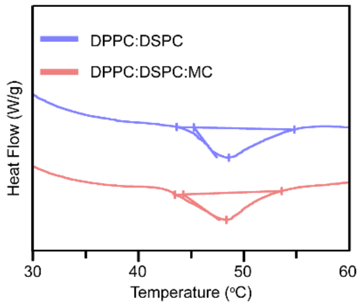


**Figure S3.** Differential scanning calorimetry thermograms of TSLs and MC-T (MC containing TSLs) at a heating rate of 10 °C $min^{-1}$, which was measured using a method described previously.[2]

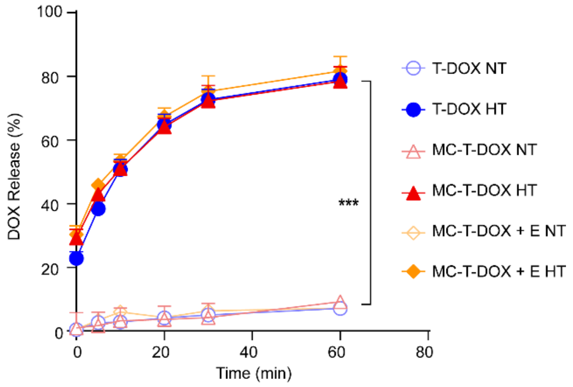


**Figure S4.** Temperature-dependent release profile of DOX from T-DOX, MC-T-DOX or MC-T-DOX + E in HEPES containing 90% fetal bovine serum. "E" indicates MT1-MMP. "MC-T-DOX + E" indicates that MC-T-DOX that had incubated with MT1-MMP. NT: normothermia, 37 °C; HT: hyperthermia, 42 °C. Data are presented as means ± SD ($n$ = 3). ***$p$ < 0.001 among the marked groups using nonparametric two-tailed analysis of variance.

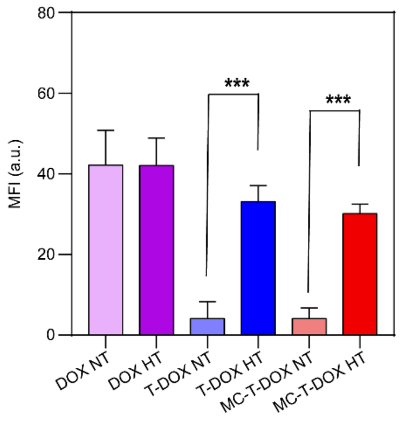


**Figure S5.** Mean DOX fluorescence intensity of BxPC-3 cells after treatment with the indicated formulations, as determined using flow cytometric examination. Data are presented as means ± SD ($n$ = 3). ***$p < 0.001$ among the marked groups using nonparametric two-tailed analysis of variance.

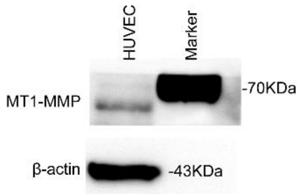


**Figure S6.** MT1-MMP expression in HUVECs analyzed by Western blot assay.

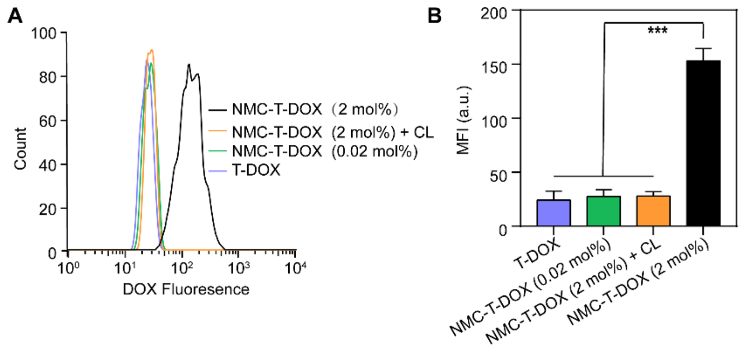


**Figure S7.** (A) Representative flow cytometry histograms of BxPC-3 cells treated with NMC-T-DOX (2 mol%), NMC-T-DOX (2 mol%) + CL, NMC-T-DOX (0.02 mol%), and T-DOX. (B) Mean fluorescence intensity from panel A. CL denotes cilengitide. Data are presented as means ± SD ($n$ = 3). ***$p < 0.001$ among the marked groups using nonparametric two-tailed analysis of variance.

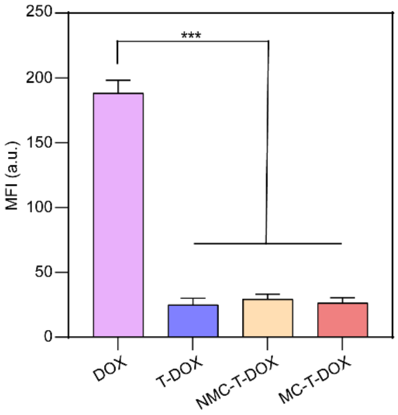


**Figure S8.** Mean DOX fluorescence intensity of HUVEC cells after treatment with the indicated formulations, as determined using flow cytometric examination. Data are presented as means ± SD ($n$ = 3). ***$p$ < 0.001 among the marked groups using nonparametric two-tailed analysis of variance.

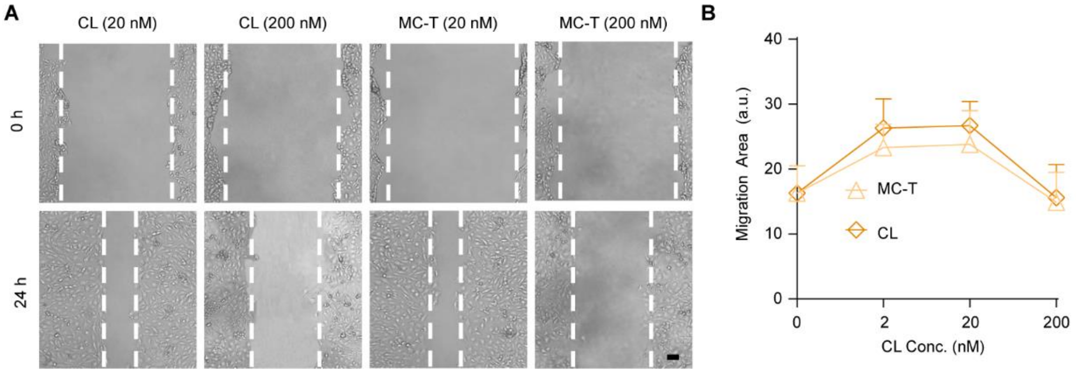


**Figure S9.** (A) Representative micrographs of HUVEC migration at 0 h and 24 h and (B) the plot of migration area vs. cilengitide concentration for cilengitide (CL) or MC-T (MC containing TSLs) after 24 h of migration. Scale bar: 200 μm.

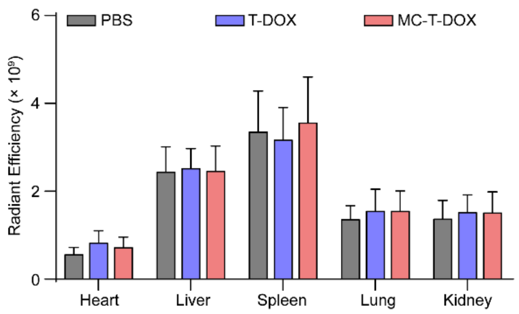


**Figure S10.** Semiquantitative fluorescence intensities of the excised hearts, livers, spleens, lungs or kidneys. Briefly, BxPC-3-bearing mice were treated with PBS, T-DOX, or MC-T-DOX for 3 cycles in a time interval of 4 days. On day 12, the mice were injected with DiR-labeled TSLs. At 24 h postinjection, the animals were sacrificed and the major tissues were excised and imaged. Data are presented as mean ± SD (*n* = 3).

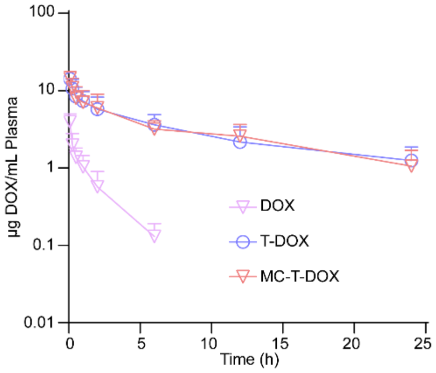


**Figure S11.** Plasma concentration-time profiles of DOX in tumor-free Sprague Dawley rats after intravenous injection of DOX, T-DOX or MC-T-DOX at an identical DOX dose of 3 mg $kg^{-1}$.

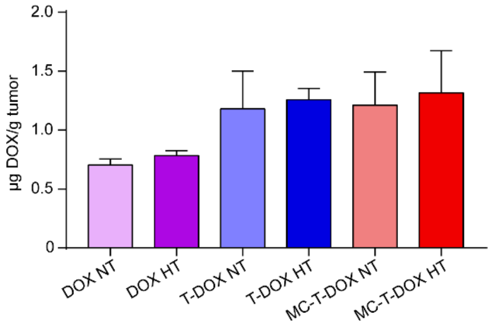


**Figure S12.** Effect of heat on tumor accumulation of MC-T-DOX. Bilateral subcutaneous BxPC-3-bearing mice were intravenously injected with DOX, T-DOX, or MC-T-DOX at a DOX dose of 3 mg $kg^{-1}$. At 2 h postinjection, the right-side tumors were subjected to mild hyperthermia (42 °C) for 1 h, and the left-side tumors were not heated. Afterwards, the animals were sacrificed and the amounts of DOX in tumors were quantitatively analyzed. Data are presented as mean ± SD ($n$ = 3).

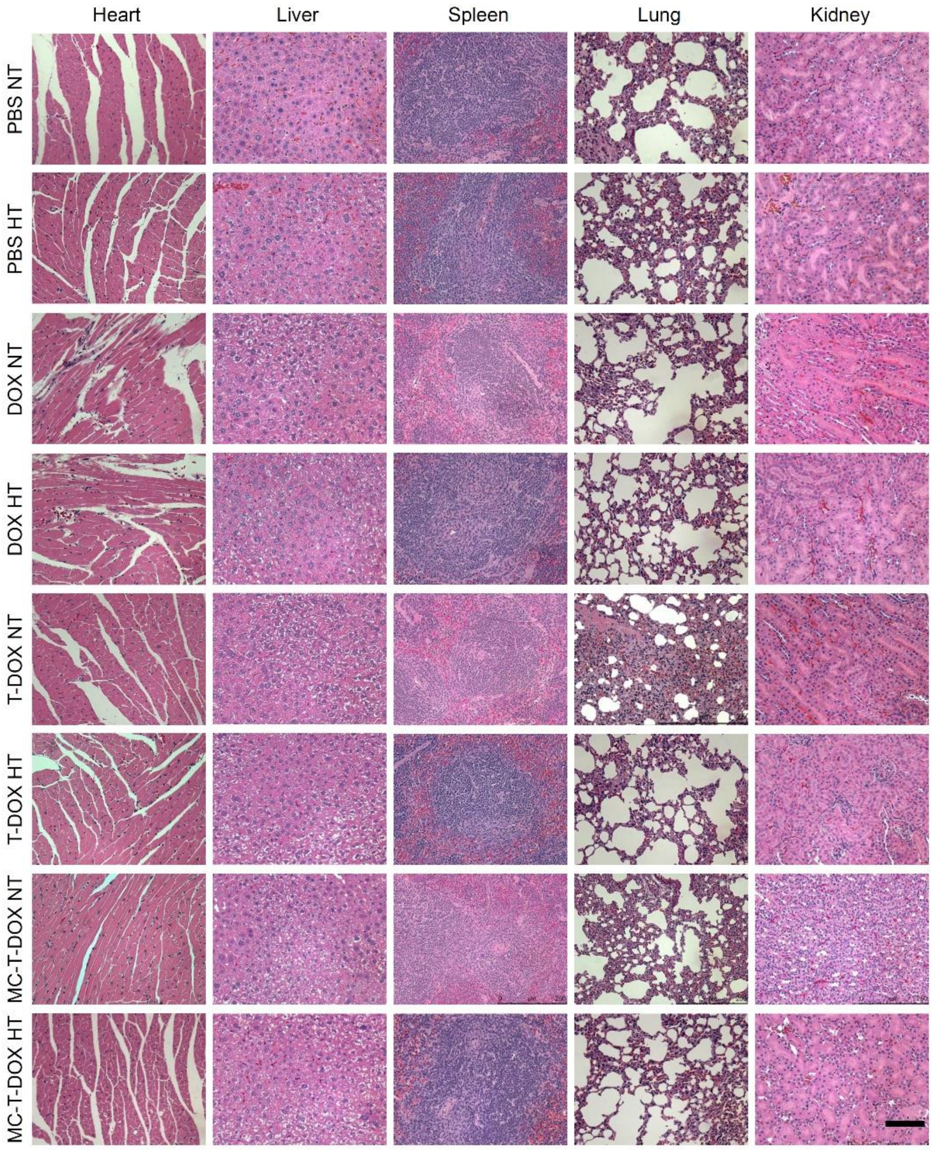


**Figure S13.** Hematoxylin-eosin (H&E) staining of the major organs (hearts, livers, spleens, lungs and kidneys) collected at the end of the antitumor therapeutic study. For the hyperthermia (HT) groups, at 2 h postinjection, the xenografts were subjected to mild hyperthermia (42 °C) in a heated water bath for 1 h, while for the normothermia (NT) groups, the xenografts were not heated. Scale bar: 100 μm.

**References:**

[1] L. Li, T. L. M. Ten Hagen, M. Hossann, R. Süss, G. C. van Rhoon, A. M. M. Eggermont, D. Haemmerich, G. A. Koning, *J. Controlled Release* **2013**, *168*, 142.

[2] Y. Wei, Y. Wang, D. Xia, S. Guo, F. Wang, X. Zhang, Y. Gan, *ACS Appl. Mater. Interfaces* **2017**, *9*, 25138.
